# *Boesenbergia rotunda*: From Ethnomedicine to Drug Discovery

**DOI:** 10.1155/2012/473637

**Published:** 2012-11-27

**Authors:** Tan Eng-Chong, Lee Yean-Kee, Chee Chin-Fei, Heh Choon-Han, Wong Sher-Ming, Christina Thio Li-Ping, Foo Gen-Teck, Norzulaani Khalid, Noorsaadah Abd Rahman, Saiful Anuar Karsani, Shatrah Othman, Rozana Othman, Rohana Yusof

**Affiliations:** ^1^Department of Molecular Medicine, Faculty of Medicine, University of Malaya, 50603 Kuala Lumpur, Malaysia; ^2^Drug Design and Development Research Group (DDDRG), University of Malaya, 50603 Kuala Lumpur, Malaysia; ^3^Department of Chemistry, Faculty of Science, University of Malaya, 50603 Kuala Lumpur, Malaysia; ^4^Division of Pharmacy, School of Pharmacy and Health Sciences, International Medical University (IMU), Bukit Jalil, 57000 Kuala Lumpur, Malaysia; ^5^Department of Pharmacy, Faculty of Medicine, University of Malaya, 50603 Kuala Lumpur, Malaysia; ^6^Institute of Biological Sciences, Faculty of Science, University of Malaya, 50603 Kuala Lumpur, Malaysia; ^7^Biotechnology and Bioproduct Research Cluster (UMBIO), University of Malaya, 50603 Kuala Lumpur, Malaysia

## Abstract

*Boesenbergia rotunda* is a herb from the *Boesenbergia* genera under the Zingiberaceae family. *B. rotunda* is widely found in Asian countries where it is commonly used as a food ingredient and in ethnomedicinal preparations. The popularity of its ethnomedicinal usage has drawn the attention of scientists worldwide to further investigate its medicinal properties. Advancement in drug design and discovery research has led to the development of synthetic drugs from *B. rotunda* metabolites via bioinformatics and medicinal chemistry studies. Furthermore, with the advent of genomics, transcriptomics, proteomics, and metabolomics, new insights on the biosynthetic pathways of *B. rotunda* metabolites can be elucidated, enabling researchers to predict the potential bioactive compounds responsible for the medicinal properties of the plant. The vast biological activities exhibited by the compounds obtained from *B. rotunda* warrant further investigation through studies such as drug discovery, polypharmacology, and drug delivery using nanotechnology.

## 1. Introduction

### 1.1. *Boesenbergia rotunda* and its Morphology

*Boesenbergia rotunda* is a ginger species that grows in Southeast Asia, India, Sri Lanka, and Southern China. This species belongs to the family of Zingiberaceae. It was previously categorised under the *Kaempferia* genus by Baker. However, it is now classified under the *Boesenbergia* genus [1]. There are many local synonyms to its name, such as Chinese keys or Fingerroot in English, “Temu Kunci” in Malay and Krachai or Krachai-Dang in Thailand. This plant has 8 different botanical names which are *Boesenbergia cochinchinensis* (Gagnep.) Loes., *Boesenbergia pandurata *(Roxb.) Schltr.,* Curcuma rotunda *L., *Gastrochilus panduratus* (Roxb.) Ridl.*, Gastrochilus rotundus* (L.) Alston,* Kaempferia cochinchinensis* Gagnep., *Kaempferia ovate* Roscoe, and *Kaempferia pandurata *Roxb.; nonetheless it is currently known as* Boesenbergia rotunda *(L.) Mansf. [2].

**Figure 1:**
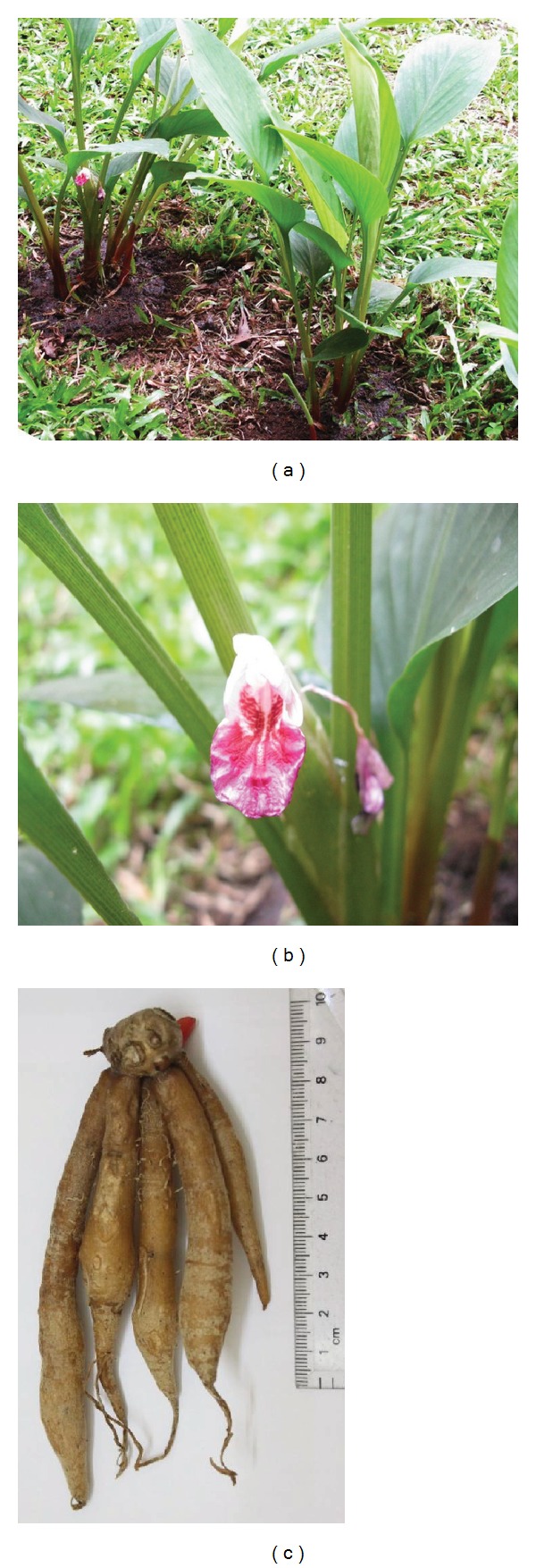
The morphology of yellow rhizome *B. rotunda*. Whole plant of *B. rotunda* (a), flower (b), and rhizomes (c) of *B. rotunda*.

*Boesenbergia* is amongst the genera facing problems in taxonomy classification due to its heterogeneous external morphology. Despite this problem, current molecular studies such as chloroplast DNA, nuclear internal transcribed spacer (ITS), random amplified polymorphic DNA (RAPD), plastid regions, pollen-based classifications, amplified fragment length polymorphism (AFLP), and single strand conformation polymorphism (SSCP) have made the classification possible [3–7]. Eksomtramage and Boontum [8] have distinguished between *B. rotunda* and *B.*  aff.  *rotunda* which have similar morphology through chromosome analysis. The chromosome count (2*n*) for *B. rotunda* and *B.*  aff.  *rotunda* was found to be 36 and 20, respectively. On the other hand, the chromosome count for *B. longipes, B. plicata,* and *B. xiphostachya* was 22. These results were reconfirmed in 2002 [8, 9]. The morphology of this ginger species has been well characterized. It is a small perennial plant of about 15–40 cm in height. Its leaves are broad and light green while the leaf sheath is red. Each shoot consists of 3–5 elliptic-oblong-red sheathed leaves of about 7–9 cm in width and 10–20 cm in length. The underground portion of the plant consists of a small globular shaped central subterraneous rhizome (1.5–2.0 cm in diameter) from which several slender and long tubers sprout all in the same direction like the fingers of a hand, thus the common name fingerroot. The tubers are about 1.0–1.5 cm thick in diameter and 5–10 cm long. The tissue of the tuber is looser, softer, and more watery than the central rhizome. Both the colour of the central rhizome and the tubers are dependent on the variety of *B. rotunda*. The yellow variety produces bright yellow rhizomes, while other varieties produce red and black rhizomes. They are strongly aromatic although different from each other. The flowers are scarlet and bloom throughout the year in tropical countries. These beautiful flowers are usually hidden at the base of the foliage, making them unnoticeable. The morphology of *B. rotunda* is shown in Figure 1 [10, 11].

### 1.2. Ethnomedicinal Functions of *B. rotunda*

*B. rotunda* is a common edible ingredient in many Asian countries such as Malaysia, Thailand, Indonesia, India, and China. It is normally cultivated at small home ranches and used as a condiment in food such as curry and soup due to its aromatic flavour, which promotes appetite. This herbal plant is also used as a traditional medicine to treat illnesses such as rheumatism, muscle pain, febrifuge, gout, gastrointestinal disorders, flatulence, carminative, stomach ache, dyspepsia, and peptic ulcer. In Indonesia, *B. rotunda* is typically used to prepare “jamu,” a popular traditional tonic for women after childbirth as well as a beauty aid for teenage girls and to prevent leukorrhea [12]. The fresh rhizomes are used to treat inflammatory diseases, such as dental caries, dermatitis, dry cough and cold, tooth and gum diseases, swelling, wounds, diarrhoea, and dysentery, and as a diuretic [13, 14]. Besides, it is also used as an antifungal and antiparasitic agent to heal fungal infections and eradicate helminth or round worms in human intestine, respectively, as well as an antiscabies agent to relieve skin itchiness from mite bites [15]. Referred to as “Thai ginseng” in Thailand, this plant is used as an aphrodisiac among Thai folk. In addition, consumption of its leaves has been shown to alleviate food allergies and poisoning. Moreover, it has been used as self-medication by AIDS patients in Thailand. Despite the lack of scientific evidence to prove the ethnomedicinal uses of this ginger, the success of current biological researches could potentially explain the significance of its traditional usage.

### 1.3. Pharmaceutical and Medicinal Functions of *B. rotunda*

Over the years, using various approaches and technologies, researchers have successfully isolated an array of bioactive compounds from *B. rotunda* Table 1. Nearly a hundred of compounds were isolated and elucidated, ranging from the flavonoid derivatives, chalcone derivatives, esters, kawains, terpenes and terpenoids (see supplementary data 1 available online at doi:10.1155/2012/473637 for more details about the chemical structures of these isolated compounds). These compounds have shown to exhibit great medicinal potential (Table 2), which will be described in more details on the following subtopics

## 2. Toxicity Tests

Given the high consumption of *B. rotunda* and the fact that its safety has not been scientifically established, recent studies have been focused on investigating the acute toxicity of several extracts *in vivo *to determine the safety of this plant for consumption. Saraithong et al. [40] demonstrated that the ethanolic extract of *B. rotunda *was safe for consumption as *in vivo* studies showed no significant changes in the body weight of *B. rotunda* fed rats. Furthermore, all haematological and histopathological parameters used to evaluate the toxicity effect did not show any adverse changes [40]. Meanwhile, Charoensin et al. [41] reported that two bioactive compounds from *B. rotunda*, pinostrobin and pinocembrin, exhibited no mutagenic effect or toxicity towards Wister rats, further confirming the safety of its consumption [41].

## 3. Antimicrobial Activities

### 3.1. Anti-*Helicobacter pylori* Activity

*Helicobacter pylori* is a prominent Gram-negative bacteria that causes gastritis, dyspepsia, and peptic ulcer and has been linked to the development of gastric and colon cancer. Widespread claims of the antimicrobial activities of *B. rotunda* prompted scientists to further evaluate the potential of this plant in preventing the infection of *H. pylori.* Pinostrobin and red oil from the roots of *B. rotunda* were found to exhibit anti-*H. pylori *activities against several different isolates of *H. pylori* [42]. The minimum inhibitory concentration (MIC) for pinostrobin and the red oil were 125 *μ*g/mL and 150 *μ*g/mL, respectively, which were comparable to the positive control, clarithromycin (120 *μ*g/mL); the minimum bactericidal concentration (MBC) was determined to be 150 *μ*g/mL and 175 *μ*g/mL, respectively. Interestingly, while both extracts inhibited *H. pylori* growth after 3 days, the growth of other bacteria was inhibited by pinostrobin, but not red oil [42]. The ethanolic extract of *B. rotunda* was also reported to significantly reduce *H. pylori* infection in Mongolian gerbils. Treated gerbils showed reduced acute and chronic inflammation when fed with *B. rotunda* 3 weeks before being challenged with *H. pylori*, and 6 weeks after. Therefore, flavonoid components of *B. rotunda* could potentially serve as potential drug candidate for inhibition of *H. pylori* infection [43].

### 3.2. Pathogenic and Spoilage Bacteria Inhibition Activities

Pathogenic bacteria are a group of bacteria that induce diseases in humans and plants. Spoilage bacteria are another group of bacteria that cause food spoilage through fermentation and decomposition of food products. There has been a rising concern pertaining to food safety and diseases caused by these pathogenic microorganisms, and hence, a renewed interest in finding new antimicrobial agents to combat these pathogens. In 2006, Pattaratanawadee et al. studied the antimicrobial activity of different extracts from four Zingiberaceae species, namely, *Zingiber officinale *Rosc (ginger),* Curcuma longa *Linn. (tumeric),* Alpinia galangal* Stuntz (galangal), and *Boesenbergia pandurata *Schltr (fingerroot), on different strains of pathogenic bacteria (*Salmonella enterica* serotype Typhimurium: 2380, 2486, 2576, 2582, *Escherichia coli *O157 : H7, *Listeria monocytogenes*: 101, 108, 310, Scott A, and V7, *Bacillus cereus,* and *Staphylococcus aureus*) and spoilage bacteria (*Lactobacillus plantarum*: PD26 and PD110 and *Lactobacillus cellobiosus*: RE33, PD32, PD40, PD55 and PD112). The MIC value for all plant extracts was between 8 and 10% (v/v) against Gram-negative bacteria, while fingerroot showed the highest inhibitory activity against three pathogenic bacteria; *L. monocytogenes*, *B. cereus,* and *S. Aureus,* with an MIC value of 0.2–0.4% (v/v). Meanwhile, galangal extract showed strongest inhibition against spoilage bacteria *L. plantarum* and *L. cellobiosus,* with an MIC value of 4% (v/v). Both galangal (at 8% (v/v)) and fingerroot (at 10% (v/v)) exhibited bactericidal effect against *E. coli* population (log cfu/mL) at 36 and 9 hours, respectively, while 8% (v/v) turmeric extract showed bacteriostatic effect [44]. Panduratin A of *B. rotunda* was also found to possess antimicrobial activity against *Staphylococcus* strains with MIC_50_ of 0.5 *μ*g/mL and MIC_90_ of 1 *μ*g/mL, both comparable to the most potent antibiotic, vancomycin [45].

### 3.3. Anti-Amoebic Activity for HIV Patients

Amoebiasis is an infection caused by the amoeba *Entamoeba histolytica*, which typically causes acute and chronic diarrhoea in HIV patients, regardless of whether they have AIDS. The use of local medicinal plants to treat this disease is popular due in part to its safety, ease of access and availability at low cost. A Previous study on thirty nine extracts from twelve local herbs reported that seven of the plant herbs, *Murraya paniculata*, *Zingiber zerumbet, Alpinia galanga*, *Barleria lupulina*, *B. rotunda*, *Piper betle* and *Piper chaba,* showed potent inhibition against three *E. histolytica* strains; HTH-56: MUTM and strain HM1: IMSS. The amoebas were cultured at 37°C for 24 hours with the plant extracts at a concentration of 1000 *μ*g/mL, and the results were determined by using inverted microscope. Based on the scoring standard of 1 to 4 with 1 indicating the highest inhibition and 4 showing no inhibition, all the seven plants scored a 1. The amoebic inhibition activities for these plants were further tested whereby extracts with IC_50_ < 100 *μ*g/mL were deemed active. The chloroform extracts of *A. galangal *(IC_50_ 55.2 *μ*g/mL), *B. lupulina* (IC_50_ 78.5 *μ*g/mL), *B. rotunda* (IC_50_ 45.8 *μ*g/mL), *P. betle* (IC_50_ 91.1 *μ*g/mL) and *P. chaba *(IC_50_ 71.4 *μ*g/mL) and the methanol extract of *B. rotunda* (IC_50_ 57.6 *μ*g/mL) were all found to be active in inhibiting propagation of amoebas [46].

## 4. Antiparasitic Activity

The inhibitory activity of *B. rotunda* against *Giardia lamblia*, a protozoan parasite that causes giardiasis, was previously demonstrated by Sawangjaroen and colleague in 2005. Giardiasis is the inflammation of the small intestine which causes diarrhoea and nutrient deficiencies, although this parasitic infection can cause chronic diarrhoea in HIV patients. The chloroform extracts of *B. rotunda* and five other herbs, which were *Alpinia galanga*, *Eclipta prostrata*, *Piper betle*, *Piper chaba*, *Zingiber zerumbet*, and the methanol extracts of *B. rotunda* and *E. prostrata* had an IC_50_ value between 20 *μ*g/mL and 100 *μ*g/mL. *A. galanga* (chloroform extract) had the highest inhibition activity with an MIC and IC_50_ values of 125 *μ*g/mL and 37.73 *μ*g/mL, respectively [47]. Although the effect of these plant extracts paled in comparison to metronidazole, a commercial antibiotic with an IC_50_ value of 0.48 *μ*g/mL, they are still considered as potential bioactive compounds that could prevent giardiasis.

## 5. Oral Infections

### 5.1. Inhibition of Biofilm Formation by Oral Pathogens

Biofilm formation on teeth surfaces is caused by multiple species of oral bacteria, the primary colonisers being mutant *Streptococci* [72]. Biofilm formation is associated with several acute and chronic infections such as dental caries, gingivitis, and periodontitis and potentially contributes to antibiotic treatment failure against *Streptococcus pyogenes *[48]. In 2008, Limsuwan and Voravuthikunchai demonstrated that the extracts from *B. rotunda*, *Eleutherine americana,* and *Rhodomyrtus tomentosa* exhibited antibiofilm activity towards *S. pyogenes *at subinhibitory concentrations (1/32–1/2 MIC) for *E. americana *(7.81–125 *μ*g/mL), *R. tomentosa *(0.24–7.81 *μ*g/mL), and *B. rotunda* (1/2 MIC of 7.8 *μ*g/mL). Antiquorum sensing test revealed that *B. rotunda* showed no inhibition activity, while *R. tomentosa* displayed strongest antiquorum effect followed by *E. americana* with moderate effect. However, microbial adhesion to hydrocarbon assay showed no changes in the cell-surface hydrophobicity of treated bacteria [48].

The following year, Yanti et al. [49] reported the anti-biofilm property of *B. rotunda *extracted panduratin A, which was found to prevent and reduce the spread of multispecies oral bacteria in human mouth. The MIC of panduratin A was determined using the Clinical and Laboratory Standards Institute (CLSI) broth microdilution assay. Mucin-mixed panduratin A at concentrations between 0.5 and 40 *μ*g/mL was coated on 96-well plates, followed by inoculation of three multispecies bacteria, *Streptococcus mutans*, *Streptococcus sanguis,* and *Actinomyces viscosus*, and incubated overnight at 37°C to allow biofilm formation. Biofilm reduction effect was determined by further treating the bacteria with different concentrations of panduratin A (0.2–10 *μ*g/mL) for up to 60 mins. Panduratin A exhibited bacteria reduction effect at MIC of 1 *μ*g/mL and bactericidal effect against multispecies planktonic cells at 2X MIC, 8 hours after treatment. Reduction of biofilm formation was >50% at 8X MIC, whereas mass reduction of biofilm was observed within 15 mins at a concentration of 10 *μ*g/mL [49]. These results suggested that panduratin A can potentially be used to prevent colonisation of multispecies bacteria, under a dose-dependent manner, and that its effect is equal to commercially available synthetic drugs such as chlorhexidine gluconate [49].

### 5.2. Antiperiodontitis Activity of *B. rotunda* Extract

A study conducted by Yanti et al. [49] on the ethanolic extract of *B. rotunda* revealed the suppressive effect of this extract on the expression of matrix metalloproteinases (MMPs) 2 and 9, both of which are overexpressed by gingival fibroblasts that are activated by *Porphyromonas gingivalis* during chronic periodontitis. They first described the inhibition of RNA and protein expression of MMP-9 by the *B. rotunda* ethanolic extract, which were 45% and 52%, respectively. Inhibition of MMP-9 was found to occur through downregulation of mitogen activated protein kinases (MAPK) phosphorylation (ERK1/2, p38, and JNK phosphorylation), thereby reducing the expression of Elk1, c-Jun, and c-Fos transcriptional factors. MMP-9 gene-regulating factors, AP-1 and NF-*κ*B, were also blocked [49].

In 2010, Yanti et al. further demonstrated the decrease in RNA and protein expression of MMP-2 in *P. gingivalis* supernatant-treated human gingival fibroblast-1 (HGF-1), and MMP-9 in *P. gingivalis* supernatant-treated oral epidermoid cells (KB cells) in a dose-dependent manner, upon treatment with the ethanolic extract of *B. rotunda* at 2 *μ*g/mL, 5 *μ*g/mL, and 10 *μ*g/mL. Suppression of MMP-2 was found to be mediated by downregulation of c-Jun N-terminal kinase (JNK) and cyclic adenosine monophosphate (cAMP) response element-binding (CREB) signalling pathways [50].

### 5.3. Inhibition of *Candida albicans*

*C. albicans *is a diploid fungus responsible for oral thrush or oral candidiasis and is a common infection observed in HIV patients. Several studies have demonstrated the potential antifungal role of *B. rotunda* in inhibiting *C. albicans *growth. A study conducted by Cheeptham and Towers [51] revealed the antifungal activity of *B. rotunda* ethanolic extract against *C. albicans* as well as *A. fumigatus*. The activation of the antimicrobial activity was found to be light mediated, as treatment performed in the dark showed no fungal inhibition [51]. In 2010, Taweechaisupapong et al. studied the antioral pathogen activities of the oil and 95% ethanolic extracts of *B. rotunda* and *Piper sarmentosum*. The extracts were screened against 4 oral pathogens, namely, *Streptococcus mutans, Lactobacillus sp*., *Aggregatibacter actinomycetemcomitans, *and* C. albican*. Results from their study showed that *B. rotunda* oil extract is a potent inhibitor against all these pathogens, as compared to *B. rotunda* ethanolic extract, and *P. sarmentosum* oil and ethanolic extracts. The MIC for *S. mutans*, *Lactobacillus sp.*, *A. actinomycetemcomitans,* and *C. albicans *was 2.0, 1.0, 0.5, and 0.5 mg/mL, respectively, for *B. rotunda* oil extract, while *P. sarmentosum* oil did not show any inhibition. In fact, *B. rotunda* oil extract showed faster killing activity towards *C. albicans* than the commercial drug, nystatin, in a time-kill curve study. Fungistatic activity was observed at concentrations of 1 and 1.5 times the MIC, whereas fungicidal effect was found at concentrations of 2 and 2.5 times the MIC, with a reduction of more than 3 log_10_ CFU/mL after 60 and 44 mins incubation, respectively [52]. These studies show that *B. rotunda* could be a potentially good source of antimicrobial agents in inhibiting oral microbes and fungal infections.

### 5.4. Anticariogenic

Dental caries or tooth decay (cavity) is a common disease caused by the oral bacteria *Streptococcus mutans* and *Lactobacillus*. These acid-producing bacteria cause damage to the tooth in the presence of fermentable carbohydrates such as sucrose and fructose, which wash away the mineral fluoride from the tooth, often resulting in tooth ache, or to a severe extent, death. The anticariogenic activity of *B. rotunda *was first described in 2004 by Hwang and colleagues. Antibacterial and bactericidal activities of *B. rotunda* methanolic extract were determined by well diffusion and viable cell count methods, respectively. Antibacterial study revealed a dose-dependent increase in the diameter of inhibition zones, with concentrations of 1 mg/mL, 10 mg/mL, and 20 mg/mL producing inhibition zones of 11 mm, 13 mm,and 14 mm in diameter, respectively. *B. rotunda* extract also showed rapid bactericidal activity against *S. mutans* in 2 mins at a concentration of 50 *μ*g/mL, rendering it practically important, given that application in toothpaste and mouthwash should be fast and effective within minutes [53].

Further isolation and purification of *B. rotunda* rhizomes yielded isopanduratin A, which was identified through ^1^HNMR. This compound conferred inhibitory properties against *S. mutans*, having an MIC value of 4 mg/L, which was lower than some natural anticariogenic agents such as green tea extract (125 mg/L) and eucalyptol (500 mg/L). At 20 mg/L, the bacteria were completely inactivated within 1 min. This compound also showed similar inhibitory activity towards *S. sobrinus*, *S. sanguinis, *and *S. salivarius *at an MIC of 4 mg/L. Microscopic observation through transmission of electron microscope revealed the destruction of the bacterial cell wall and cytoplasmic membrane detachment after treatment with 10 mg/L isopanduratin A, suggesting the potential application of isopanduratin A as a natural anticariogenic agent to prevent cariogenic effect on teeth [54].

### 5.5. Candidal Adhesion Inhibitor

Candidal adhesion is an essential mechanism for *Candida* species to adhere to the oral surfaces to colonise the mouth and cause oral diseases. Sroisiri and Boonyanit [55] recently reported that the rhizome extract of *B. rotunda* could inhibit the adhesion of *Candida* species on the denture acrylic surfaces in a dose-dependent manner. Pretreatment of the dentures with *B. rotunda* extract at concentrations of 25, 50 and 100 mg/mL significantly inhibited candidal adhesion by approximately 47%, 66%, and 74%, respectively. Ergo, it is theorised that candidal adhesion can possibly be reduced by soaking acrylic dentures in *B. rotunda* rhizome extract for 30 mins [55].

### 5.6. Antihalitosis

Halistosis (or bad breath) is a condition where the mouth produces an unpleasant odor when exhaling. This situation is typically associated with oral conditions such as gum diseases and oral hygiene. It can also be caused by the odour from esophagus, tonsil, nose, and stomach. Hwang et al. [56] have patented the optimum oral wash formulation of antihalitosis, containing panduratin derivatives from *B. rotunda*, that could reduce the effect of halitosis by 70–90% [56].

## 6. Inhibition of Biofilm Formation by Intestinal Pathogens

Given the positive findings regarding the inhibitory activity of *B. rotunda* against biofilm formation of several oral pathogens, Rukayadi et al. further investigated its inhibitory properties against enterococcal biofilm formation. *Enterococci*, particularly *E. faecalis* and *E. faecium*, are common Gram-positive cocci found typically in the intestinal tract and are known to cause intestinal and urinary infections. By employing the CSLI guidelines, the MIC of panduratin A was found to be 2 *μ*g/mL while the MBC that effectively killed all the *enterococci* isolates was 8 *μ*g/mL. At 4X MIC, the compound caused bactericidal effect after 30 mins of incubation, as determined by time-kill curve study. The MIC of panduratin A was also lower than that of other commercial antibacterial agents such as ampicillin (256 *μ*g/mL), tetracycline (64 *μ*g/mL), gentamycin (512 *μ*g/mL), and erythromycin (256 *μ*g/mL), indicating that it is more potent than commonly administered antibiotics [57].

## 7. Antioxidant Activities

### 7.1. Inhibition of Lipid Peroxidation in Brain

Lipid peroxidation is the oxidative degradation of lipid, which is a damaging process as generation of peroxidation products leads to the spread of free-radical reactions. Free-radicals are known causes of pathological disturbances such as atherosclerosis and myocardial infarction. The antioxidant activity of *B. rotunda* rhizome was recently demonstrated by Shindo and colleagues [29], who studied the free-radical scavenging activities of six bioactive compounds from *B. rotunda* rhizome extract; (−)-panduratin A, (−)-4-hydroxypanduratin A, 2′,6′-dihydroxy-4′-methoxychalcone, 2′,4′-dihydroxy-6′-methoxychalcone, 5-hydroxy-7-methoxyflavanone, and 5,7-dihydroxyflavanone, on rat brain homogenate model. Lipid peroxidation activity on these compounds, extracted using CH_2_Cl_2_–MeOH (1 : 1) and purified by using hexene-EtOAc (5 : 1) in silica gel column, revealed two potent inhibitors, (−)-panduratin A (IC_50_ 15 *μ*M) and (−)-4-hydroxypanduratin A (IC_50_ 4.5 *μ*M). Neuroprotective effect of these compounds was also demonstrated through L-glutamate toxicity study. Four compounds, (−)-panduratin A, 2′,6′-dihydroxy-4′-methoxychalcone, 2′,4′-dihydroxy-6′-methoxychalcone, and (−)-4-hydroxypanduratin A, showed high neuroprotective effect with an effective concentration (EC_50_) of 13 *μ*M, 37 *μ*M, 48 *μ*M, and 14 *μ*M, respectively, as compared to (+)-catechin (160 *μ*M). The side chain structures of (−)-panduratin A and (−)-4-hydroxypanduratin A are likely causes for the higher inhibitory activity of these 2 compounds [29].

### 7.2. Inhibition of Oxidative Damages by *tert*-Butylhydroperoxide (*t*-BHP)

Sohn et al. [58] reported the protective effects of panduratin A against *t*-BHP, an organic hydroperoxidant that initiates lipid peroxidation through its metabolism to free-radical intermediates, causing oxidative damage to cells. MTT cell viability assay showed a decrement in HepG2 cell growth inhibition by *t*-BHP, whereas fluorometric measurement revealed a dose-dependent reduction in malondialdehyde (MDA) formation and glutathione (GSH) depletion, upon treatment with panduratin A. Intracellular reactive oxygen species (ROS) production was also reduced from 665 ± 11.79 (mean ± standard deviation) to 170 ± 30.62 when treated with 15 *μ*M of the compound, further implying the potential application of this compound as a natural antioxidant [58].

## 8. Antiulcer Effect

*B. rotunda* is also used as a traditional medicine to treat ulcer by local communities in Thailand and Indonesia. The antiulcer effect of *B. rotunda* methanolic extract and its pure compound, pinostrobin, was recently explored by Abdelwahab and coworkers [59]. *B. rotunda* extract and pinostrobin exhibited cytoprotective effects on ulcer-induced rats, as evidenced by the reduction in ulcer area and mucosal content. In addition, submucosal edema and leukocytes infiltration were significantly reduced or prevented. The antioxidant activity of pinostrobin was proven through its ability to reduce the level of thiobarbituric acid reactive substances (TBARS) and through ferric reducing antioxidant power (FRAP) assay which gave a value of 116.11 ± 0.004 (mean ± standard deviation) [59].

## 9. Obesity Treatment

Obesity is a metabolic disorder that poses a global threat to humans. Caused by fat accumulation due to improper energy balance and lipid metabolism, obesity can cause liver and cardiovascular diseases. Panduratin A, previously determined to be a novel natural AMP-activated protein kinase (AMPK) activator, was studied in attempts to decipher the regulatory mechanisms involved in AMPK-PPAR*α*/*δ* signalling. AMPK is an enzyme that regulates cellular energy through activation of LBK1 and Ca^2+^/calmodulin-dependent protein kinase kinase **β**  (CaMKK**β**). The activation of AMPK will increase the fatty acid oxidation by activating fatty acid oxidation-related genes. This process will prevent lipid synthesis via reduction of sterol regulatory element-binding protein-1c (SREBP-1c) and PPAR*γ* phosphorylation. When 50 mg/kg/day of panduratin A was applied, AMPK signalling was found to be stimulated, nuclear translocation of AMPK*α*2 induced, followed by activation of PPAR*α*/*δ*, with LKB1 being the key mediator of these effects. Activation of PPAR*α*/*δ* increased fatty acid oxidation, resulting in weight loss, and reduced fat pad mass as observed in the *in vivo *obese mouse model. Moreover, these mice showed reduction in fatty liver and an improvement in the serum lipid profiles. Myofibre proportion and mitochondria content in muscles were significantly increased, enhancing running endurance [60]. Taken together, these results exemplify the usefulness of panduratin A in treating obesity and associated metabolic disorders.

## 10. Anti-Mutagenic Effect

Mutagens such as mutagenic heterocyclic amines [3-amino-1,4-dimethyl-*5H*-pyrido [*4,3*-*b*]indole (Trp-P-1), 3-amino-1-methyl-5*H*-pyrido [4,3*-b*]indole (Trp-P-2) and 2-amino-1-methyl-6 phenylimidazo [4,5-*b*]pyridine (PhIP)] are found in cooked food or processed meat and fish and can cause chromosomal and DNA damages, increasing the risk towards diseases such as cancer. In 2001, Trakoontivakorn et al., in the collaboration with a Japanese research group, tested the antimutagenic properties of six extracted bioactive compounds from *B. rotunda* rhizome against Trp-P-1, Trp-P-2, and PhIP in *Salmonella typhimurium* TA98. All six compounds, pinocembrin chalcone, cardamonin, pinocembrin, pinostrobin, 4-hydroxypanduratin A, and panduratin A, exhibited potent antimutagenic effects, having IC_50_ values of 5.2 ± 0.4, 5.9 ± 0.7, 6.9 ± 0.8, 5.3 ± 1.0, 12.7 ± 0.7, and 12.1 ± 0.8 *μ*M, respectively, with Trp-P-1 as the mutagen. Similar inhibition was also observed with Trp-2-P and PhIP, with the N-hydroxylation of Trp-2-P being strongly inhibited [23]. This shows that the purified flavonoids, chalcones, and cyclohexenyl chalcone derivatives (CCD) from *B. rotunda* strongly hinder mutagenesis.

## 11. Antitumour Necrosis Factor Alpha (Anti-TNF-**α**)

Tumour necrosis factor-*α* is a pleiotropic inflammatory cytokine that plays an imperative role in immune response to bacterial, fungal, and viral infections, as well as in the necrosis of specific tumours. Extensive production of TNF-*α* gives rise to health problems such as autoimmune or chronic inflammatory disorders, for instance, rheumatic arthritis. Morikawa et al. [35] recently reported that prenylchalcones and prenylflavanones, extracted from *B. rotunda*, could inhibit TNF-*α*. They found that (+)-krachaizin B, (−)-krachaizin B, (+)-4-hydroxypanduratin A, (−)-4-hydroxypanduratin A, (+)-isopanduratin A,(−)-isopanduratin A, alpinetin, cardamonin, and 2,6-dihydroxy-4-methoxydihydrochalcone showed strong inhibition towards TNF-*α* on L292 cells at 10 *μ*M, whereas (+)-krachaizin B, (−)-krachaizin B, (+)-panduratin A, (−)-panduratin A, (+)-4-hydroxypanduratin A, (−)-isopanduratin A, and geranyl-2,4-dihydroxy-6-phenylbenzoate strongly inhibited the activity of aminopeptidase N [35].

## 12. Anticancer/Antitumour

### 12.1. Breast Cancer and Colon Cancer Prevention

Breast and colon cancers are among the leading causes of cancer deaths worldwide. Despite the extensive ongoing research in finding an effective treatment regime or anticancer drug to fight these diseases, researchers are still far from uncovering a breakthrough, due in part to the lack of knowledge on the physiology of these cancers. Nevertheless, many current researches are still focused on natural plant herbs as potential targets in anticancer drug development, with *B. rotunda* being amongst them. Kirana et al. [61] screened through eleven species of Zingiberaceae and found *B. rotunda* and *Zingiber aromaticum* to exhibit the highest inhibition towards MCF-7 breast cancer and human HT-29 colon cancer cell growth, with the IC_50_ values being 21.3 ± 0.3 *μ*g/mL and 32.5 ± 1.5 *μ*g/mL, and 20.2 ± 1.8 *μ*g/mL and 11.8 ± 1.0 *μ*g/mL, respectively. Morphological studies of the cells suggested death by apoptosis, as evidenced by the appearance of membrane blebs, nuclear condensation, and formation of apoptotic bodies [61]. Further evaluation of another compound, panduratin A, on the same cell lines revealed potent inhibitory properties as well. The IC_50_ values for MCF-7 and HT-29 cells were determined to be 3.75 *μ*g/mL and 6.56 *μ*g/mL, respectively. Cell cycle and proliferation studies showed that 71% of the cells were arrested at G0/G1 after treatment with panduratin A, as compared to 33% for untreated cells. Additionally, animal study showed that this compound was nontoxic to the rats as no obvious weight loss was observed, and the aberrant crypt foci formation, although reduced, was not significantly different as compared to the control [62]. In 2004, Zaeoung et al. reported cytotoxic activities of *B. rotunda* volatile oils against breast cancer MCF-7 (IC_50_  31.7 ± 5.4 *μ*g/mL) and LS174T colon cancer (IC_50_  12.0 ± 1.6 *μ*g/mL) cell lines [28].

In a separate study, Jing and colleagues demonstrated that *B. rotunda* possessed the strongest inhibitory effects against CaOV_3_ ovarian cancer (IC_50_ 71 ± 1.41 *μ*g/mL), breast cancer MDA-MB-231 (IC_50_  66.5 ± 2.12 *μ*g/mL), MCF-7 (IC_50_ 51 *μ*g/mL), HeLa cervical cancer (IC_50_  65.5 ± 2.12 *μ*g/mL), and HT-29 colon cancer (IC_50_  52 ± 4.24 *μ*g/mL) cell growth as compared to three other *Boesenbergia* species, *B. pulchella *var *attenuate, *and *B. armeniaca *[39]. Cell cycle analysis on treated MCF-7 cells revealed *B. rotunda* to effectively arrest cells at sub-G1 phase, whereas *B. pulchella *var *attenuate *arrested the cell cycle at G2/M phase [73].

### 12.2. Inhibition of Prostate Cancers

In 2006, Yun et al. demonstrated that treatment with panduratin A could inhibit the growth of prostate cancer cell lines (PC3 and DU145) in a time and dose-dependent manner, with IC_50_ values of 13.5 and 14 *μ*M for PC3 and DU145 cells, respectively. Immunoflourescence assay showed that panduratin A triggered the induction of apoptosis in both cell lines, through the inhibition of apoptotic-related procaspases 3, 6, 8, and 9. Apoptosis was suggested to occur via the mitochondrial-dependent pathway, as evidenced by the increase of Bax : Bcl-2 ratio by 6- and 15-fold for PC3 and DU145 cells, respectively, and the upregulation of Fas death receptor and TNF-related apoptosis-inducing ligand (TRAIL). Cell cycle analysis revealed cell cycle arrest at G2/M phase in a dose-dependent manner. Moreover, immunoblot analysis showed induction of p21^WAF/Cip1^ and p27^Kip1^, and downregulation of cdks 2, 4, and 6, and cyclins D1 and E [64]. These findings suggest that panduratin A could be a potential therapeutic agent against prostate cancer.

### 12.3. Antilung Cancer

Aside from the anticancer properties towards breast, colon, and prostate cancer, panduratin A also exhibited inhibitory activities against A549 human nonsmall cell lung cancer cells. The IC_50_ value of this compound was 4.4 *μ*g/mL, as determined by MTT assay, whereas cell cycle analysis via flow cytometer showed cell cycle arrest at mitotic/M phase. Panduratin A also acted as nuclear factor kappa beta (NF-*κ*B) inhibitor, as treatment at apoptosis-inducing concentration was found to inhibit translocation of NF-*κ*B from cytoplasm to nuclei by the activation of tumour necrosis factor alpha (TNF-*α*) [65].

### 12.4. Antileukemia

*B. rotunda* rhizome has been suggested to possess antileukemic property, as demonstrated by Sukari and colleagues [34]. Cytotoxicity assay on *B. rotunda* extracts and five flavonoid derivatives, pinostrobin, pinocembrin, alpinetin, cardamonin, and boesenbergin A, revealed that most extracts and pure compounds were able to inhibit the growth of HL-60 cancer cell line, particularly the chloroform extract and boesenbergin A [34].

## 13. Antifungal Activities

### 13.1. Antifungal Activities against AIDS-Related Fungal Infections

AIDS is one of the major infectious diseases in Thailand and is most commonly transmitted sexually. HIV patients are susceptible to fungal infections such as candidiasis by *Candida* species, cryptococcosis by *Cryptococcus* species, and histoplasmosis by *Histoplasma capsulatum*, and traditional herbs are typically sought after as natural treatment. Phongpaichit et al. [66] reported that the chloroform extract from rhizome of *B. rotunda *could inhibit the propagation of *Candida neoformans *and *Microsporum gypseum *but showed low effect against *C. albicans *by using antifungal assay. From the disc diffusion assay, the plant extract of *B. rotunda* showed the smallest diameter range as compared to other plant extracts, with inhibition zones of 8.0 ± 0.1–0.6 mm and ±9.0 mm in diameter for *C. albicans* and *Cryptococcus neoformans*, respectively. The MIC for the chloroform extract of *B. rotunda* was 64 *μ*g/mL against *C. neoformans* and *M. gypseum, *which was determined to be the lowest and most active among the plant extracts in inhibiting fungal growth. The methanolic extract, on the other hand, had an MIC of 128 *μ*g/mL against *C. neoformans*. Conversely, both extracts had little inhibition against *C. albicans *(MIC > 512 *μ*g/mL) [66].

### 13.2. Inhibition of Spoilage and Aflatoxin Producing Fungi

Food spoilage due to spoilage fungi, such as aflatoxin fungi, is a major concern among consumers. Pattaratanawadee et al. [44] showed that the ethanolic extract of *B. rotunda* could inhibit spoilage fungi activities (*Aspergillus flavus*, *Aspergillus niger*, *Aspergillus parasiticus, and Fusarium oxysporum*) with MICs of >10% (v/v), 8% (v/v), 10% (v/v), and <8% (v/v), respectively [44]. This shows that *B. rotunda* is a good choice to inhibit the growth of certain spoilage fungi.

## 14. Inhibition of Ca^2+^ Signal in Yeast Model

Ca^2+^ signalling is one of the crucial physiological pathways in most living organisms, playing imperative roles in regulating diverse cellular processes such as T-cell activation and apoptosis. Extensively studied in the yeast *Saccharomyces cerevisiae*, Ca^2+^ signalling is implicated in the regulation of G2/M cell cycle progression, and inappropriate activation of this signalling pathway can cause physiological and developmental defects. Wangkangwan et al. [67] reported that the bioactive compounds from the crude rhizome extract of *B. rotunda* could inhibit Ca^2+^ signalling in *S. cerevisiae* mutant *zds1*Δ strain. Further purification of the crude extract led to isolation of three compounds, namely, pinostrobin, alpinetin, and pinocembrin chalcone. Yeast proliferation assay showed significant inhibition by pinostrobin (low cytotoxicity), alpinetin (high cytotoxicity), and pinocembrin chalcone (high cytotoxicity) with MICs of <0.5, 1, and 0.5, respectively. Given the low toxicity of pinostrobin at 1 mM, it was further subjected to biochemical studies and was found to relieve hyperactivation of Ca^2+^ signals in yeast, which is responsible for abnormal morphology and growth arrest at G2 phase. Flow cytometry analysis revealed that treatment with 1 mM pinostrobin prevented G2 arrest of yeast cells, and normal morphology characterised by equal nuclei distribution and the absence of abnormal budding was observed [67].

## 15. Antiviral Activities

### 15.1. Anti-HIV-1 Protease Activity

The HIV-1 protease (aspartyl protease class), a highly conserved protein component for viral maturation, propagation, and infectivity in the human body, is a promising drug target currently under extensive research for the development of drugs and therapeutics to combat HIV/AIDS. The anti-HIV protease activity of *B. rotunda *rhizomes was previously characterised by Cheenpracha et al. [30]. Purification of methanolic extract of *B. rotunda* rhizomes yielded cyclohexenyl chalcones panduratin A, panduratin C, hydroxypanduratin A, and chalcone derivatives, helichrysetin, 2′,4′,6′-trihydroxyhydrochalcone, and uvangoletin. Their results showed that hydroxypanduratin A and panduratin A exhibited high inhibition, with IC_50_ values of 5.6 *μ*M and 18.7 *μ*M, respectively, as compared to other bioactive compounds which showed weaker inhibition. Structure activity relationship (SAR) study revealed that the effectiveness of the HIV-1 protease inhibition is related to the hydroxylation and prenylation of chalcones [30]. In a separate study, Tewtrakul and colleagues [26] investigated the anti-HIV protease activity of chloroform, methanol and water extracts of several traditional herbs used by Thai locals as self-medication for AIDS. The chloroform extract of *B. rotunda* exhibited the most potent inhibition against HIV-1 protease (64.92 ± 4.75%), followed by methanolic extract with 51.92 ± 0.22% inhibition as compared to other plant species [26]. In another study, four flavonoids, namely, pinostrobin, pinocembrin, cardamonin, and alpinetin, were isolated from the ethanolic extract of *B. rotunda* rhizomes. Antiviral assay showed that cardamonin exhibited the highest inhibition of 75.11 ± 1.44% with an IC_50_ value of 31.0 *μ*g/mL [27]. Therefore, cardamonin, panduratin A, and hydroxypanduratin A are potential drug targets to inhibit HIV-1 protease activity.

### 15.2. Inhibition of Dengue NS2B/NS3 Protease

Dengue virus serotype-2 (Flaviviridae family) is one of the four dengue serotypes responsible for causing dengue fever, dengue haemorrhagic fever, and dengue shock syndrome worldwide. To date, there is no commercial vaccine available to circumvent the spread of the virus. Research is still underway to develop an effective vaccine or drug and natural compounds are currently among the important antiviral resources.

In 2006, Tan et al. studied the inhibition activity of CCD and flavonoids from *B. rotunda* against dengue NS2B/NS3 protease cleavage. Based on their results, CCD such as 4-hydroxpanduratin A and panduratin A showed the strongest inhibition, with inhibition constant, K_*i*__,_ values of 21 *μ*M and 25 *μ*M, respectively, as compared to pinocembrin, pinostrobin, cardamonin, and alpinetin. 4-Hydroxypanduratin A had higher inhibition percentage (78.1 ± 0.1%) than panduratin A (66.7 ± 0.1%) at a low concentration of 80 ppm, when using substrate 1 [tert-butyloxycarbonyl-glycyl-L-arginyl-L-arginine-4-methylcoumaryl-7-amide (Boc-Gly-Arg-Arg-MCA)] to cleave the protease. Although pinocembrin and cardamonin did not show high inhibition, combination of both compounds, however, managed to inhibit the NS2B/NS3 protease activity by 81.8 ± 0.3% at 400 ppm. For substrate 2 (Boc-Gln-Arg-Arg-MCA), 4-hydroxypanduratin A still showed the highest inhibition at 90% at a concentration of 120 ppm, compared to pinostrobin and panduratin A, which nonetheless showed good inhibition as well [74]. Thus, these compounds may serve as potential inhibitors for dengue-2 NS2B/NS3 protease.

### 15.3. Antifoot and Mouth Disease Virus (Anti-FMDV)

Foot and mouth disease (FMD) is caused by the infection of a picornavirus called the Foot and Mouth Disease virus (FMDV). FMDV infects cloven-hoofed animals such as bovids, and also sometimes humans. In Thailand, *B. rotunda* is typically used as an anti-FMDV natural herb by local folks. In 2007, a group of Thailand researchers investigated the antiviral activity of 42 local herb extracts against FMDV, 24 of which exhibited anti-FMDV activity. While the most potent extracts were the crude extracts of *Morinda elliptica* (TCID_50_ of 1 × 10^3.65^) and *Morinda citrifolia* (TCID_50_ of 1 × 10^3.35^) at concentrations of 0.39 *μ*g/*μ*L and 0.19 *μ*g/*μ*L, respectively, *B. rotunda* also showed anti-FMDV activity with TCID_50_ 1 × 10^2.14^ at 0.012 *μ*g/*μ*L concentration [68].

## 16. Anti-Inflammatory

Inflammation is a biological process that is activated in response to extracellular stimulants such as pathogens and chemicals, to mitigate the effects or heal the organism. *B. rotunda* has been traditionally used in treating several inflammatory-related diseases such as gout, allergy, and peptic ulcer. Scientific research has proven the anti-inflammatory properties of this plant, as discussed below.

### 16.1. TPA-Induced Ear Oedema

The inflammatory activity of *B*. *rotunda* was studied on rats with 12-O-tetradecanoyl phorbol-13-acetate (TPA)-induced ear oedema. (−)-Hydroxypanduratin A and (−)-panduratin A, isolated from the chloroform extract of B. rotunda red rhizomes, were found to exhibit strong anti-inflammatory activities compared to the other extracted compounds, sakuranetin, pinostrobin, pinocembrin, and dihydro-5,6-dehydrokawain. The IC_50_ values for (−)-Hydroxypanduratin A and (−)-panduratin A were 84 *μ*g/ear and 12 *μ*g/ear, respectively. (−)-hydroxypanduratin A inhibited TPA-induced ear oedema by 73% at 2000 *μ*g/ear concentration and 10 hrs treatment, whereas 94% inhibition was obtained with (−)-panduratin A treatment at the same concentration and incubation time point. Ear oedema thickness was also significantly reduced to 48 ± 6 *μ*m from 94 ± 6 *μ*m and 11 ± 4 *μ*m from 81 ± 8 *μ*m for (−)-hydroxy panduratin A and (−)-panduratin A treated rats, respectively [25]. Further investigation is warranted to study the mechanisms of action and targets of both compounds with regards to their anti-inflammatory activities.

### 16.2. Inflammation by Nitric Oxide (NO), Prostaglandin E2 (PGE2), and Tumour Necrosis Factor Alpha (TNF-*α*)

In 2009, Tewtrakul et al. reported that the extracts of *Kaempferia parviflora* and *B. rotunda* exhibited anti-inflammatory effects through the inhibition of nitric oxide (NO), prostaglandin E2 (PGE2), and tumour necrosis factor alpha (TNF-*α*). NO acts as an inflammatory intermediator within the human metabolic processes, defending against intracellular and extracellular stimulants. Excess of this molecule; however, will induce pathogenesis in cells and form reactive free-radical upon reaction with other radicals. These reactive radicals cause direct damage to the function of normal cells. PGE2 and TNF-*α* are also inflammatory inter-mediators involved in inflammation and carcinogenesis. Panduratin A and hydroxypanduratin A, which were purified from *B. rotunda* methanolic extract, showed strong inhibitory activity against NO, with IC_50_ values of 5.3 *μ*M and 13.3 *μ*M, respectively, compared to the most potent compound of *K. parviflora *which was 5-hydroxy-3,7,3′,4′-tetramethoxyflavone (IC_50_ 16.1 *μ*M). High inhibition against PGE2 production was observed, whereby both panduratin A and hydroxypanduratin A had IC_50_ values of 10.5 *μ*M and 12.3 *μ*M, respectively, which was comparable to that of 5-hydroxy-3,7,3′,4′-tetramethoxyflavone (IC_50_ 16.3 *μ*M). Conversely, only a moderate inhibitory activity on TNF-*α* was observed for all three compounds, panduratin A (IC_50_ 60.3 *μ*M), hydroxypanduratin A (IC_50_ 57.3 *μ*M), and 5-hydroxy-3,7,3′,4′-tetramethoxyflavone (IC_50_ > 100 *μ*M) [69].

### 16.3. Anti-Inflammatory Effect Caused by *Opisthorchis viverrini*

*Opisthorchis viverrini *is a parasite from the Opisthorchiidae family that causes cholangiocarcinoma in humans. It is disseminated through consumption of raw and uncooked fish and can cause inflammation during the infection. Boonjaraspinyo et al. [70] reported that *B. rotunda* rhizome could inhibit inflammation caused by *O. viverrini* and induced by N-nitrosodimethylamine administration (NDMA) in rats. Histopathological study showed that the liver tissues of normal and *B. rotunda*-treated rats exhibited similar morphology (no inflammation observed) compared to NDMA-treated (higher cytotoxicity effect) and *O. viverrini*-infected rats which showed inflammation around the hepatic bile ducts after one month. Upon treatment with *B. rotunda* plant extract, *O. viverrini*-infected and NDMA-treated liver cells showed a reduction in the inflammatory cells surrounding the hepatic bile ducts, which correlated with the decrement in the levels of serum alanine transaminase and direct bilirubin, but not of alkaline phosphatase, which remained the same level as untreated group [70].

## 17. Inhibition of Platelet-Activating Factor (PAF) Receptor Binding Effect

PAF is a phospholipid mediator that is involved in many negative physiological functions and pathological conditions such as bronchoconstriction-induced asthma, hyperacute organ-transplant rejection, gastrointestinal ulceration, thrombosis, and allergic reaction. Jantan et al. [71] showed that the PAF receptor binding effect could be inhibited by using local medicinal herb extracts obtained in Malaysia. They successfully identified eleven plant extracts (*Alpinia galangal*, *B. rotunda*, *Curcuma anthorrhiza*, *Curcuma aeruginosa*, *Zingiber officinale*, *Zingiber zerumbet*, *Cinnamomum altissimum*, *Cinnamomum pubescens*, *Goniothalamus malayanus*, *Momordica charantia,* and *Piper aduncum*) that significantly reduced the binding effect of PAF on rabbit platelets (IC_50_ values ranging from 1.2 to18.2 *μ*g/mL) with more than 60% inhibition. The strongest inhibition effect was shown by *Z. zerumbet* with IC_50_ of 1.2 ± 2.0 *μ*g/mL (96.4 ± 1.4% inhibition), followed by *B. rotunda* with IC_50_ of 8.6 ± 2.6 *μ*g/mL (80.4 ± 4.1% inhibition), when compared with cedrol (control) which showed 85.2 ± 2.4% inhibition at IC_50_ value of 2.4 ± 1.3 *μ*g/mL [71]. Therefore, these plant extracts can be used to potentially treat PAF-mediated diseases.

## 18. Wound Healing Properties

 Ethanolic extracts of *B. rotunda* rhizome have been shown to accelerate wound healing in rats [75]. Visually, it was shown that wounds dressed with rhizomes extract and Intrasite gel significantly healed earlier than those treated with vehicle. Histological analysis of healed wounds dressed with rhizomes extract showed comparatively less scar width at wound closure and healed wound contained less inflammatory cells and more collagen with angiogenesis compared to wounds dressed with vehicle only.

From the empirical results mentioned previously, *B. rotunda* contains potential bioactive compounds with multiple medicinal properties that can prevent, mitigate, or treat various diseases as well as prevent them from spreading. Table 2 summarises the inhibition activities of various *B. rotunda* extracts towards diseases investigated and reported by scientists worldwide.

## 19. Current Research on *B. rotunda*

### 19.1. Tissue Culture and Metabolite Engineering

*In vitro* culture provides an immediate source of compounds in a sustainable approach and as study subjects for molecular biology, crop improvements, and genetics studies. Furthermore, it offers a feasible platform with defined, controllable chemical and physical conditions for metabolite engineering. Rao and Ravishankar [76] described a nonredundant list of plant-derived pharmaceuticals and highlighted the importance of the availability of contaminant-free samples, optimum multiplication, and regeneration protocol for metabolite engineering studies. With that, uniform quality and yield of the compounds could be achieved without problem with variation due to seasonal and geographical reason [76].

Optimization of the culture conditions for *B. rotunda* has been carried out by Tan et al. [77]. By manipulating the concentration of the plant growth regulator, 2,4-dichlorophenoxyacetic acid (2,4-D), in Murashige and Skoog [78] nutrient medium, Tan et al. [77] have successfully induced somatic embryo from meristematic tissue of the young plant. Somatic embryogenesis is a process whereby a somatic cell undergoes development analogous to the zygotic embryo and regenerates into a clonal plant. With the medium formulation, 23.3 ± 4.3% of embryogenic callus formed, and the plantlet regeneration rate was 6.6 ± 0.1 plantlets from a callus aggregate of 1 cm diameter. This finding has made somatic embryogenesis a model system to mass propagate identical cell/tissue samples of *B. rotunda *for metabolite engineering.

Besides somatic embryogenesis, another route of plantlets regeneration of *B. rotunda *has been investigated. Yusuf et al. [79] reported a mass production method from young shoot bud in MS medium with sucrose (30.0 g/L), gelrite (2.0 g/L), different concentrations of 6-benzylaminopurine (BAP), and *α*-naphthaleneacetic acid (NAA). Combination of 2.0 mg/L BAP and 0.5 mg/L NAA was found to be the best treatment for callus induction while multiple shoots (90%, 5 shoots/explant) were induced from day 10 to 14. In all treatments, roots spontaneously grew after 10–14 days. Acclimatization of the *in vitro* plantlets in soil was successfully performed.

Cell culture in liquid suspension is also an important alternative source for metabolite engineering, mainly owing to the fast propagating rate and ease of scaling-up [80]. Furthermore, secondary metabolites accumulation in suspension culture can be manipulated and enhanced by external chemical and physical treatments with simplicity. Direct contact between cell and nutrients or treatment agents present in the liquid media permits a quick response of the cell.

Chemical treatments such as nutrient level, sucrose level, plant growth regulator (PGR), precursor, and substrate feeding are the common methods facilitating the enhancement of compound yield in suspension cultures. By modifying growth-associated factors such as sucrose level, nutrient level especially phosphate and nitrogen composition, and the addition of different types of PGR, productivity of both biomass and compounds could be increased [81]. On the other hand, relative productivity of compounds per dry weight of cell biomass could be enhanced with nongrowth-related precursors and elicitors.

Precursors are substrates or intermediates found in the biosynthesis pathways from which the secondary metabolites formed [82], while elicitors are physiological stimuli from various abiotic or biotic sources that trigger secondary metabolites accumulation [83]. Metal salts and inorganic ions are common abiotic elicitors used in treatments of plant cell cultures for compounds enhancement. Biotic elicitors include microbial cells or components of microbial cell and even plant signalling molecules or compounds such as yeast extract (YE), salicylic acid, and methyl jasmonate.

Zhao et al. [84] examined the effects of four classes of biotic and abiotic elicitors, heavy metal ions (cobalt, silver nitrate, and cadmium chloride), polysaccharides from YE and chitosan, salicylic acid, methyl jasmonate, and sorbitol on the production of diterpenoid tanshinones in *Salvia miltiorrhiza* cell culture. Amongst the treatments, 25 *μ*M Ag^+^ (silver nitrate), 25 *μ*M Cd^+^ (cadmium chloride), and 100 mg/L polysaccharide from YE successfully enhanced tanshinone production by more than ten fold compared to the control. Interestingly, the authors also reported suppressed cell growth with a decrease in biomass yield by about 50% in treated *S. miltiorrhiza* cell culture. In another example, treatment of *Taxus chinensis* cells with Ag^+^ following adaptation in chitosan through several subcultures resulted in a 4.6-fold increase in paclitaxel production compared to the unadapted cells [85]. The authors also suggested that the treatment using elicitors needed to be optimized and explored thoroughly for promising results.

Physical treatments such as stress factors, light sources, modification of culture environment and electric current, and Pulsed Electric Field (PEF) methods are also widely used for metabolite engineering in plant cell cultures [86]. Kaimoyo et al. successfully enhanced the production of medicarpin by 168-fold in cell suspension [87].

Cai et al. [88] examined the effects of elicitors and high hydrostatic pressure on secondary metabolism of *Vitis vinifera* suspension culture. The authors used the concentration of phenolic compounds as a measure of the secondary metabolism level and found that it was significantly higher than in the control when treated with ethephon. When the treatment was carried out with combination of ethephon and high hydrostatic pressure, extracellular phenolic acids and 3-O-glucosyl-resveratrol were increased. The results showed that hydrostatic pressure can be used for compound secretion into the liquid medium. Cai et al. [89] further explained that high hydrostatic pressure might alter the permeability of cell/membrane and cause secretion of the compounds as a defense response of plant cells. In *V. vinifera *cv. Chasselas 9 and *Vitis berlandieri* cell suspension cultures in a 2 L stirred bioreactor, 90% of the total resveratrol can be secreted into the liquid medium [90].

In conclusion, with the availability *in vitro* sources for *B. rotunda*, the study and enhancement of secondary metabolite production can be robustly performed and the bioreactor technology could be employed for maximising the yield in the near future.

### 19.2. Chemical Syntheses of Bioactive Compounds Isolated from *B. rotunda*

The syntheses of flavonoid compounds derived from *B. rotunda* have been well documented. A review on the stereoselective synthesis of flavonoids has been reported [91]. Although important progress has been made in the syntheses of these compounds, the systematic study of B. rotunda-derived flavonoids, that is, (−)-pinostrobin and (±)-panduratin A is still hampered by the inaccessibility of enantiomeric pure starting materials.

**Scheme 1:**
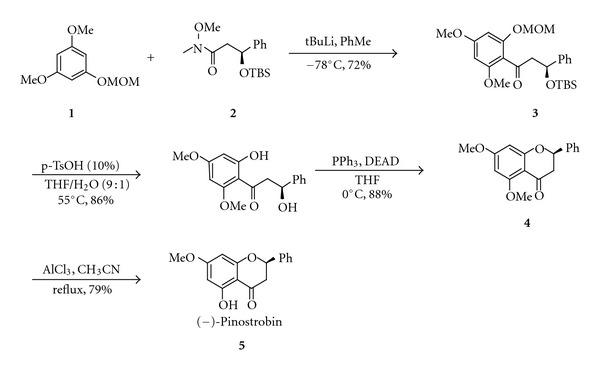


The enantioselectivity of (−)-pinostrobin was first reported by Hodgetts [92]. The method was based on an intramolecular Mitsunobu cyclisation of the chiral hydroxyphenol **3**, which was prepared from the Weinreb amide **2** and the methoxymethyl (MOM)-protected phenol **1 **(Scheme 1). Subsequent cleavage of the protecting groups on **3** allowed the intramolecular Mitsunobu cyclisation to produce methoxylated pinostrobin **4**. Finally, regioselective demethylation of **4** with aluminium chloride gave enantiomeric pure (−)-pinostrobin in 60% overall yield from **3**.

**Scheme 2:**
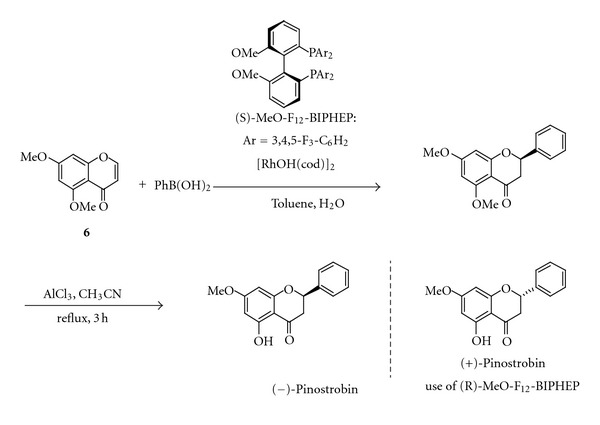


Recently Korenaga et al. have described an efficient method to synthesise (+)- and (−)-pinostrobin via rhodium-catalysed asymmetric 1,4-addition of phenylboronic acid to 5,7-dimethoxychromone **6 **(Scheme 2). The reaction in toluene proceeded smoothly at room temperature in the presence of 0.5% rhodium catalyst with electron-poor chiral diphosphine MeO-F_12_-BIPHEP ((6,6′-dimethoxybiphenyl-2,2′-diyl)bis[bis-3,4,5-trifluorophenyl)phosphine]) to produce the desired pinostrobin in high yield and high enantioselectivity (90% yield, 99.6% ee) [93].

**Scheme 3:**
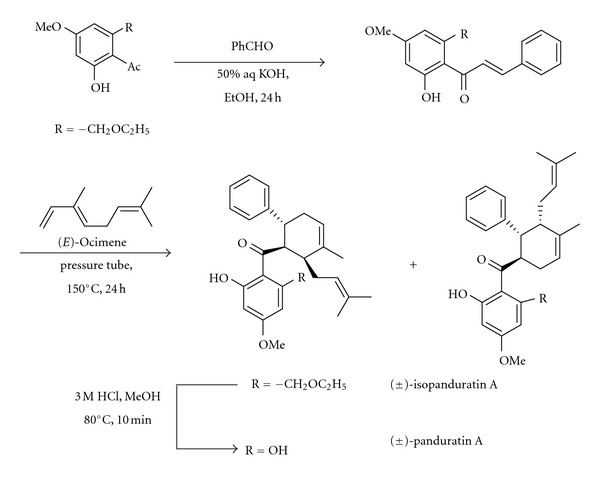


We have recently reported the synthesis of (±)-panduratin A and its regioisomer (±)-isopanduratin A in four steps from (*E*)-ocimene via a Diels-Alder cycloaddition reaction (Scheme 3) [94]. However, attempts to use the chiral auxiliaries or catalysts such as the CBS-oxazaborolidines and MacMillan's imidazolidinones for the enantioselective synthesis of panduratin A were not successful.

Kavalactones such as (+)-kavain and yangonin are the major components of kava extract (*Piper methysticum*) and have shown a wide spectrum of pharmacological activities [95–103]. Derivative of kavalactones such as 5,6-dehydrokavain has been found in *B. rotunda*. The synthesis of these six-membered unsaturated *δ*-lactones has generated a widespread interest. Various synthetic routes to racemic and enantiopure kavalactones have been studied via Aldol reaction involving cinnamaldehydes but none of them are generally applicable to the synthesis of oxygen-substituted kavalactones such as yangonin [104–117].

Amaral and coworkers described a strategy to synthesise yangonin and derivatives based on the Heck cross-coupling reaction of pyrone **7** and aryl iodides **8** (Figure 5). Reaction of the pyrone **7**, aryl iodides, and 10 mol% of tetrakis(triphenylphosphine)palladium (0) in the presence of Hünig's base in DMF under microwave irradiation (300 W) for 5.5 min produced the desired kavalactones in moderate yields [118].

**Scheme 4:**
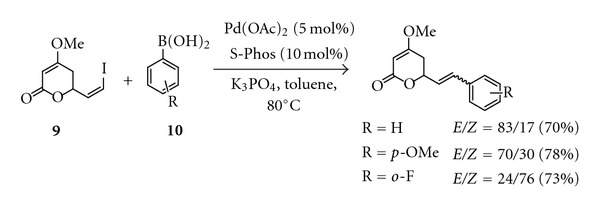


They also devised an alternative strategy to synthesise kavalactones via Suzuki-Miyaura coupling reaction between (*Z*)-6-(2-iodovinyl)-4-methoxy-5,6-dihydro-2*H*-pyran-2-one **9 **and aryl boronic acid **10** (Scheme 4). However, the reaction which catalysed by Pd(OAc)_2_ and 2-(2,6-dimethoxyphenyl) dicyclohexylphosphine (S-Phos) resulted in the formation of an *E/Z* mixture of cross-coupling products.

**Scheme 5:**
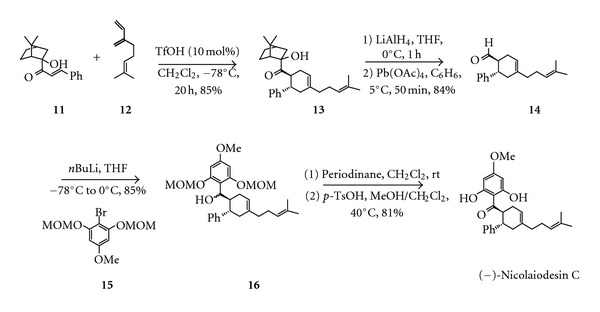


The synthesis of (−)-nicolaiodesin C (also known as (−)-krachaizin A) has been reported by Banuelos and coworkers [119]. Accordingly, the BrØnsted acid-assisted Diels-Alder reaction of (1R)-(+)-camphor-derived chiral *α*′-hydroxyenone **11** and myrcene **12** first produced an enantiopure adduct **13**. Subsequent reduction of the carbonyl group in **13** with LiAlH_4_ and oxidative cleavage of the resulting diol with Pb(OAc)_4_ provided aldehyde **14**. Addition of the lithium anion of MOM-protected methoxyresorcinol **15** to aldehyde **14** afforded the alcohol **16**. Finally, (−)-nicolaiodesin C was obtained after oxidation of the alcohol **16** with periodinane in dichloromethane and deprotection of the MOM group in acidic conditions (Scheme 5).

### 19.3. Drug Discovery through Bioinformatics

**Figure 2:**
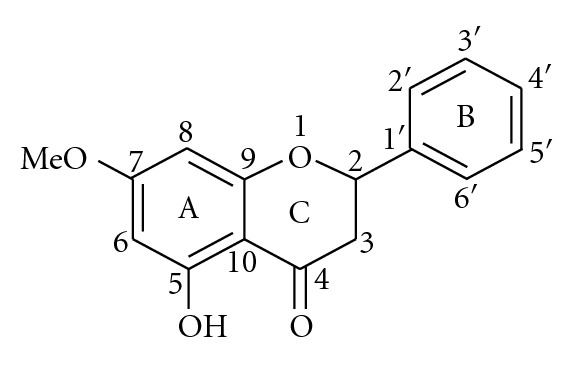
Structure of pinostrobin.

Some bioactive compounds of *B. rotunda* had been studied computationally by Othman et al. [120], Paul et al. [121], and Frimayanti et al. [122]. Three flavanones, pinostrobin, pinocembrin, and alpinetin, and four chalcones, pinostrobin chalcone, pinocembrin chalcone, and cardamonin, had been subjected to automated docking towards dengue virus type 2 NS2B/NS3 protease (Protein Data Bank id: 2FOM) to understand the interactions of these reported inhibitors [123] with the binding sites of the protease [120]. In this study, it was reported that the estimated Δ*G* (free energy of binding) for the flavanones were lower than those of their chalcone derivatives. The automated docking experiments showed that all the ligands studied did not bind to the active site of the protease, which are consistent with the bioassay results, illustrating the noncompetitive inhibitory activities for most of the ligands [74]. Through SAR analysis, it was also suggested that the higher noncompetitive inhibitory activity shown by pinostrobin compared to the other compounds could be accounted for by H-bonding interaction with the backbone carbonyl of Lys74, which is bonded to Asp75 (one of the catalytic triad residues). As shown in Figure 2, the rigid structure of flavanone, the C5 hydroxyl and C7 methoxy groups on ring A, and the phenyl ring (B) was also suggested to be important features to consider in designing new compounds with potential inhibitory activities against dengue virus type 2 NS2B/NS3 protease.

Another docking study was performed in 2011 by Frimayanti et al. using 2FOM structure, and the homology model of dengue virus type 2 NS2B/NS3 protease with reported competitive inhibitors [74], 4-hydroxypanduratin A and panduratin A from *B. rotunda* as reference compounds. The derivatives of these compounds were then used as ligands for docking, and subsequently, new competitive inhibitors were designed based on the docking result. Based on Figure 4, substitutions were performed individually on positions 1, 2, 3, 4, and 5 of the benzyl ring A of 4-hydroxypanduratin A and panduratin A. It was found that substitutions at positions 4 and 5 gave the lowest and closest energies to the reference compounds from the calculated complexation energies, and new ligands were designed by substituting various functional groups on these positions. This strategy was suggested to be an early stage drug discovery for identifying drug candidates.

**Figure 3:**
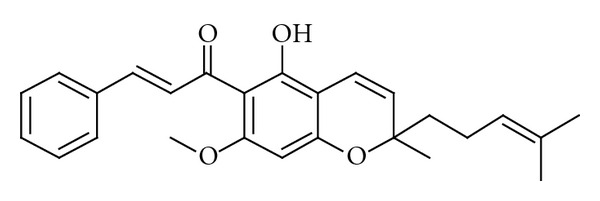
Structure of boesenbergin B.

**Figure 4:**
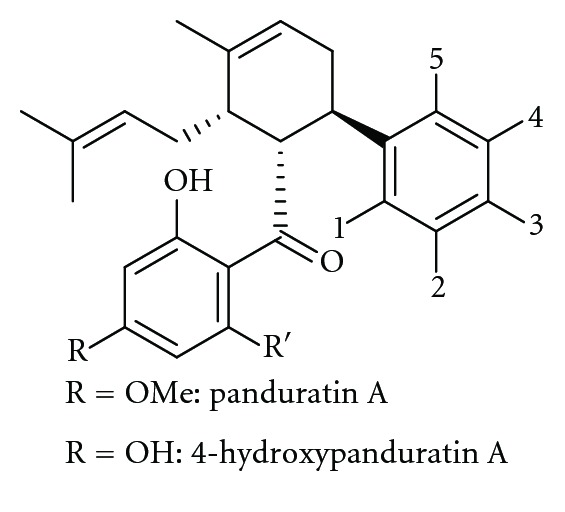
Structures of 4-hydroxypanduratin A and panduratin A.

**Figure 5:**
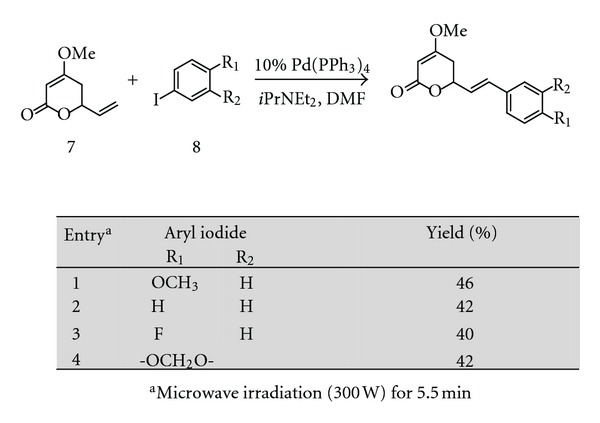
Synthesis of kavalactones via Heck cross-coupling reaction.

 In 2010 [121], Paul and Choudhury conducted molecular docking studies on the activity of naturally occurring pyranochalcones on the transcriptional regulator enzyme of *Pseudomonas putida*, a gram-negative bacteria that is resistant to antibiotics. In his studies, the HTH-type transcriptional regulator TTgR (Protein Data Bank id: 2UXI) in *P. putida *(bound with phloretin) was taken as the target for docking with pyranochalcones as ligands. One of the pyranochalcones, boesenbergin A (Figure 3), was isolated from *B. rotunda* rhizomes and reported as a highly potential candidate to be active against the multidrug resistant strain of bacteria. From the SAR analysis, the binding affinity of the pyranochalcones was found to increase with increasing number of methoxy moiety in the aromatic part of the ligands. In contrast, the effect of the methoxy moiety connected to the fused aromatic unit, seemed to be less pronounced, could be due to steric hindrance. The results are hoped to be useful in designing new series of drugs especially against the antibiotic-resistant bacteria.

## 20. Protein Profile of *B. rotunda*

Since *B. rotunda* has many medicinal uses, it is important to explore the molecular level of the biosynthesis of the targeted plant metabolites, especially panduratin A and 4-hydroxypanduratin A, that have high therapeutic values. In 2011, Chong et al. conducted a preliminary study using proteomic approaches to extract the total protein from the callus (normal and treated callus). This is the initial step to obtain the protein profile of the callus that can be used to analyse the protein expression level of the callus after treatment with phenylalanine, a precursor of phenylpropanoid biosynthesis pathway, to produce CCD, through an unknown biosynthesis pathway in *B. rotunda* [124].

## 21. Future Prospects of Plant-Based Drug

The ethnomedicinal usages of *B. rotunda*, supported by strong scientific evidence of its potential medicinal properties, clearly justify that this plant should indeed be brought to the next level of drug discovery studies, directed towards metabolomics, genomics, transcriptomics, proteomics, and bioinformatics aspects to further characterise the mechanisms and pathways that contribute to its desired properties. The wide inhibition range of *B. rotunda* against multiple diseases such as cancers, microbes, viruses, and parasites should be further explored through new drug discovery studies such as plant-derived compounds, polypharmacology, drug-DNA/protein interactions, and, specific drug stability, solubility, and delivery to the targeted organ, by using nanotechnology.

## 22. Conclusion

*B. rotunda* is a native ingredient in many Asian countries and is used as a condiment in food. It is also used as traditional medicine to treat several illnesses, consumed as traditional tonic especially after childbirth, beauty aid for teenage girls, and as a leukorrhea preventive remedy for women. Its fresh rhizomes are also used to treat inflammatory diseases, in addition to being used as an antifungal, antiparasitic, and aphrodisiac among Thai folks. Its leaves are used by locals to alleviate food allergic and poisoning. Moreover, AIDS patients self-medicate themselves with *B. rotunda* to cure the infection. With the advancement in technology, the ethnomedicinal usages of herbal plants can be explained through *in vitro* and *in vivo* studies to prove the activities of the plant extracts. The current state of research on *B. rotunda* clearly shows that the isolated bioactive compounds have high potential in treating many diseases. With the development of medicinal chemistry and bioinformatics, we are well on our way to successfully develop plant-based drugs. Molecular progressions further encourage scientists to delve deeper into the biosynthetic pathways of *B. rotunda* bioactive compounds to obtain a bigger picture of the whole process, which in turn could accelerate the development of better and stronger drugs to counter the diseases in the future.

## Supplementary Material

Chemical structure of *B. rotunda* bioactive compounds

## Figures and Tables

**Table 1:** Bioactive compounds that were extracted from the leaves, stems, and rhizomes of *B. rotunda. *

Compounds	Isolated by
5-Hydroxy-7-methoxyflavanone (pinostrobin) 5,7-Dihydroxyflavanone (pinocembrin) 2′,6′-Dihydroxy-4′-methoxychalcone (pinostrobin chalcone) 2′,4′-Dihydroxy-6-methoxychalcone (Cardamonin) Boesenbergin A	Jaipetch et al. [16]

5-Hydroxy-7-methoxyflavanone (pinostrobin) 5,7-Dimethoxyflavanone 5-Hydroxy-7-methoxyflavone 5-Hydroxy-7,4′-dimethoxyflavone 5,7-Dimethoxyflavone 5,7,4′-Trimethoxyflavone 5,7,3′,4′-Tetramethoxyflavone 5-Hydroxy-3,7-dimethoxyflavone 5-Hydroxy-3,7,4′-trimethoxyflavone 3,5,7-Trimethoxyflavone 5-Hydroxy-3,7,3′,4′-tetramethoxyflavone	Jaipetch et al. [17]

2,6-Dihydroxy-4-methoxyphenyl-[3′-methyl-2′-(3′′-methylbut-2′′-enyl)-6′-phenylcyclohex-3′-enyl]methanone (panduratin A) Rubranine Pinostrobin Boesenbergin A	Tuntiwachwuttikul et al. [18]

Boesenbergin B Panduratin A	Mahidol et al. [19]

3,5,7,3′,4′-Pentamethoxyflavone 3,5,7,4′-Tetramethoxyflavone 5-Hydroxy-7,4′-dimethoxyflavanone 2′-Hydroxy-4′,6′-dimethoxychalcone 2′-Hydroxy-4,4′,6′-trimethoxychalcone	Herunsalee et al, 1987 [20]

(±)-Panduratin B1 (±)-Panduratin B2	Pancharoen et al. [21]

Pinostrobin Pinocembrin Alpinetin Panduratin A	Tip-Pyang et al. [22]

2′,4′,6′-Trihydroxychalcone (Pinocembrin chalcone) Cardamonin Pinocembrin 5-Hydroxy-7-methoxyflavanone (2,4,6-Trihydroxyphenyl)-[3′-methyl-2′-(3′′-methylbut-2′′-enyl)-6′-phenylcyclohex-3′-enyl]methanone (4-Hydroxypanduratin A) Panduratin A	Trakoontivakorn et al. [23]

Essential Oils	
Camphor	
Linalool	
Camphene	
*α*-Pinene	
*α*-Terpineol	
*α*-Phellandrene	
*γ*-Terpinene	
Methyl 3-phenylpropionate	
Geranyl formate	
Geranyl propionate	
Geraniol	
Neral	
Myrcene	
Isoborneol	
*β*-Pinene	
Neryl acetate	
Geranial	
*β*-Thujaplicin	
(E,E)-*α*-Farnesene	
Borneol	Jantan et al. [24]
Tricyclene	
Terpinen-4-ol	
Terpinolene	
Myristicin	
AlloOcimene	
*α*-Thujene	
(Z)-*β*-Ocimene	
Sabinene	
(E)-*β*-Ocimene	
(Z)-Nerolidol	
cis-Linalool oxide	
3-Carene	
*δ*-Elemene	
(Z)-*β*-Farnesene	
*γ*-Elemene	
*β*-Elemene	

(−)-4-Hydroxypanduratin A (−)-Panduratin A 5,4′-Dihydroxy-7-methoxyflavanone (sakuranetin) 5-Hydroxy-7-methoxyflavanone 5,7-Dihydroxyflavanone Dihydro-5,6-dehydrokawain	Tuchinda et al. [25]

Pinostrobin Pinocembrin Alpinetin Cardamonin	Tewtrakul et al. [26, 27]

Camphor Geraniol Methyl cinnamate Geranial (*E*-citral)	Zaeoung et al. [28]

5-Hydroxy-7-methoxyflavanone	
(−)-Panduratin A	
5,7-Dihydroxyflavone	Shindo et al. [29]
Pinostrobin chalcone	
Cardamonin	
(−)-4-Hydroxypanduratin A	

Panduratin C	
Panduratin A	
4-Hydroxypanduratin A	Cheenpracha et al. [30]
Helichrysetin	
2′,4′,6′- Trihydroxydihydrochalcone (propiophenone)	
Uvangoletin	

Pinostrobin Pinocembrin Alpinetin Cardamonin Panduratin A 4-Hydroxypanduratin A	Tan et al. [31]

(2S)-6-Geranylpinostrobin Geranyl-2,4-dihydroxy-6-phenethylbenzoate 2′,4′-Dihydroxy-3′-(1′′-geranyl)-6′-methoxychalcone (1′R,2′S,6′R)-2-Hydroxyisopanduratin A (2R)-8-Geranylpinostrobin (±)-6-Methoxypanduratin A (2S)-7,8-Dihydro-5-hydroxy-2-methyl-2-(4′′-methyl-3′′-pentenyl)-8-phenyl-2H, 6H-benzo[1,2-b:5,4-b′]dipyran-6-one (−)-Pinostrobin Tectochrysin 5,6-Dehydrokawain Cardamonin (−)-Alpinetin Flavokawain C (−)-7,4′-Dihydroxy-5-methoxyflavanone (−)-6-Geranylpinocembrin Boesenbergin A Panduratin A Boesenbergin B (±)-Isopanduratin A1 (−)-Pinocembrin (−)-4-Hydroxypanduratin A Nicolaioidesin B Panduratin C Isopanduratin A2	Win et al. [32]

Pinostrobin Pinocembrin Alpinetin Cardamonin Boesenbergin A	Ching et al. 2007 [33]; Sukari et al. [34]

Prenylchalcones	Morikawa et al. [35]
(+)-Krachaizin A	
(−)-Krachaizin A	
(+)-Krachaizin B	
(−)-Krachaizin B	
(+)-Panduratin A	
(−)-Panduratin A	
(+)-4-Hydroxypanduratin A	
(−)-4-Hydroxypanduratin A	
(+)-Isopanduratin A	
(−)-Isopanduratin A	
Prenylflavonoids	
Rotundaflavone Ia	
Rotundaflavone Ib	
Rotundaflavone iia	
Rotundaflavone IIb	
Flavanones and Others (12)	
Pinostrobin	
Pinocembrin	
Alpinetin	
7,4′-Dihydroxy-5-methoxyflavanone	
5,7-Dihydroxy-8-geranylflavanone	
7-Methoxy-5-hydroxy-8-geranylflavanone	
Cardamonin	
2,6-Dihydroxy-4-methoxydihydrochalcone	
2,4-Dihydroxy-6-phenethyl-benzoic acid methyl ester	
Geranyl-2,4-dihydroxy-6-phenylbenzoate	
5,6-Dehydrokawain	
Geraniol	

Essential oils	Sukari et al. [36]
Monoterpenes hydrocarbons	
1,8-Cineole	
*trans*-Ocimene	
Neryl acetate	
*β*-Pinene	
Oxygenated monoterpene derivatives	
Borneol	
*β*-Citronellol	
Camphor	
Bicyclo(2.2.1)heptan-2-ol	
Methyl-n-nonanoate	
*trans*-Geraniol	
2-Cyclohexyethylacetate	
3′,4′-Dimethoxyacetophenone	
Rosephenone	
Terpinyl valerate	
*N-*Hexyl angelate	
Sesquiterpene hydrocarbons	
*trans*-Caryophyllene	
1,3-Tetradecadiene	
Miscellaneous compounds	
Guaiacol	
*N*-Hexanal	
2-Isopropyl-4,5-dimethyloxazole	
*trans*-2-Hexanyl-n-propionate	
Cyclohexyl-n-propionate	
2-N-Pyroyl-4,5-dimethylthiazole	
Ethyl benzoate	
Guaiocol n-caproate	
Geranyl benzoate	
n-Butyl-n-pentadecanoate	
n-Eicosane	
Cinnamyl cinnamate	

Panduratin B1	
Panduratin B2	
Panduratin D	
Panduratin E	Win et al. [37]
Panduratin F	
Panduratin G	
Panduratin H	
Panduratin I	

Panduratin C	
Panduratin A	
Hydroxypanduratin A	
Helichrysetin	Trewtrakul et al. 2009
Propiophenone	
Uvangoletin	
Cardamonin	

Pinostrobin	
Pinocembrin	Wangkangwan et al. [38]
Alpinetin	

Polyphenols	
Quercetin	
Kaempferol	
Naringin	Jing et al. [39]
Hesperidin	
Caffeic acid	
*ρ*-Coumaric acid	
Chlorogenic acid	

**Table 2:** Inhibitory activities exhibited by *B. rotunda* extracts.

Inhibition	Sources	References
(1) Toxicity test	Rhizomes	Saraithong et al. [40]
		Charoensin et al. [41]

(2) Antimicrobial activities		
(A) Anti-*Helicobacter pylori* activity	Rhizomes	Bhamarapravati et al. [42]
		Mahady et al. [43]
(B) Pathogenic and spoilage bacteria inhibition activities	Rhizomes	Pattaratanawadee et al. [44]
		Rukayadi et al. [45]
(C) Antiamoebic activity for HIV patients	Rhizomes	Sawangjaroen et al. [46]

(3) Antiparasitic activity		
Antigiardial activity	Rhizomes	Sawangjaroen et al. [47]

(4) Oral infections		
(A) Inhibition of biofilm formation by oral pathogens	Rhizomes	Limsuwan and Voravuthikunchai, [48]
		Yanti et al. [49]
(B) Antiperiodontitis activity of *B. rotunda* extract		Yanti et al. [49, 50]
(C) Inhibition of *Candida albicans *	Dried plant	Cheeptham and Towers, [51]
		Taweechaisupapong et al., [52]
(D) Anticariogenic	Rhizomes	Hwang et al. [53, 54]
(E) Candida adhesion inhibitor	Rhizomes	Sroisiri and Boonyanit, [55]
(F) Antihalitosis	Rhizomes	Hwang et al. (patented) [56]

(5) Inhibition of biofilm formation by intestinal pathogens	Rhizomes	Rukayadi et al. [57]

(6) Antioxidant activities		
(A) Inhibition of lipid peroxidation in brain	Rhizomes	Shindo et al. [29]
(B) Inhibition of oxidative damages by *tert*-butylhydroperoxide (*t*-BHP)	Rhizomes	Sohn et al. [58]

(7) Antiulcer effect	Rhizomes	Abdelwahab et al. [59]

(8) Obesity treatment	Rhizomes	Kim et al. [60]

(9) Antimutagenic effect	Rhizomes	Trakoontivakorn et al. [23]

(10) Antitumour necrosis factor alpha (anti-TNF-*α*)	Rhizomes	Morikawa et al. [35]

(11) Anticancer/antitumour		
(A) Breast cancer and colon cancer prevention	Roots and rhizomes	Kirana et al. [61]
		Zaeoung et al. [28]
		Kirana et al. [62]
		Ling et al. [63]
(B) Inhibition of prostate cancers	Rhizomes	Yun et al. [64]
(C) Antilung cancer	Rhizomes	Cheah et al. [65]
(D) Antileukemia	Rhizomes	Sukari et al. [34]

(12) Antifungal activities		
(A) Antifungal activities against AIDS-related fungal infections	Rhizomes	Phongpaichit et al. [66]
(B) Inhibition of spoilage and aflatoxin producing fungi	Rhizomes	Pattaratanawadee et al. [44]

(13) Inhibition of Ca^2+^ signal in yeast model	Rhizomes	Wangkangwan et al. [67]

(14) Antiviral activities		
(A) Anti-HIV-1 protease activity	Rhizomes	Tewtrakul et al. [26, 27]
		Cheenpracha et al. [30]
(B) Inhibition of dengue NS2B/NS3 protease	Rhizomes	Tan et al. 2006
(C) Antifoot and mouth disease virus	Rhizomes	Chungsamarnyart et al. [68]

(15) Anti-inflammatory		
(A) TPA-induced ear oedema	Rhizomes	Tuchinda et al. [25]
(B) Inflammation by nitric oxide (NO), prostaglandin E2 (PGE2), and tumour necrosis factor alpha (TNF-*α*)	Rhizomes	Tewtrakul et al. [69]
(C) Anti-inflammation effect caused by *Opisthorchis* *viverrini*	Rhizomes (powder)	Boonjaraspinyo et al. [70]

(16) Inhibition of platelet-activating factor (PAF) receptor binding effect	Rhizomes	Jantan et al. [71]
