# Supplementary material for: Boesenbergia rotunda: From Ethnomedicine to Drug Discovery
Source: Evid Based Complement Alternat Med. 2012 Nov 27;2012:473637. doi: 10.1155/2012/473637 (PMC3519102; doi:10.1155/2012/473637)
Supplement: Supplementary file 1 — Chemical structure of B. rotunda bioactive compounds [file 473637.f1.doc]

**Supplementary Data Table 1:** Chemical structure of *B. rotunda* bioactive compounds

Flavanones

| **Entry** | **R_1_** | **R_2_** | **R_3_** | **R_4_** | **R_5_** | **Name** |
| --- | --- | --- | --- | --- | --- | --- |
| 1 | OH | OH | H | H | H | 5,7-dihydroxyflavanone (Pinocembrin) |
| 2 | OH | OCH_3_ | H | H | OH | Sakuranetin |
| 3 | OCH_3_ | OH | H | H | H | Alpinetin |
| 4 | OH | OCH_3_ | H | H | H | Pinostrobin |
| 5 | OCH_3_ | OCH_3_ | H | H | H | 5,7-dimethoxyflavanone |
| 6 | OCH_3_ | OH | H | H | OH | 7,4’-dihydroxy-5-methoxyflavanone |
| 7 | OH | OCH_3_ |  |  | OCH_3_ | 5-hydroxy-7,4’dimethoxyflavanone |
| 8 | OH | OH |  | H | H | 5,7-dihydroxy-8-geranylflavanone |
| 9 | OH | OCH_3_ |  | H | H | 7-methoxy-5-hydroxy-8-geranylflavanone |
| 10 | OH | 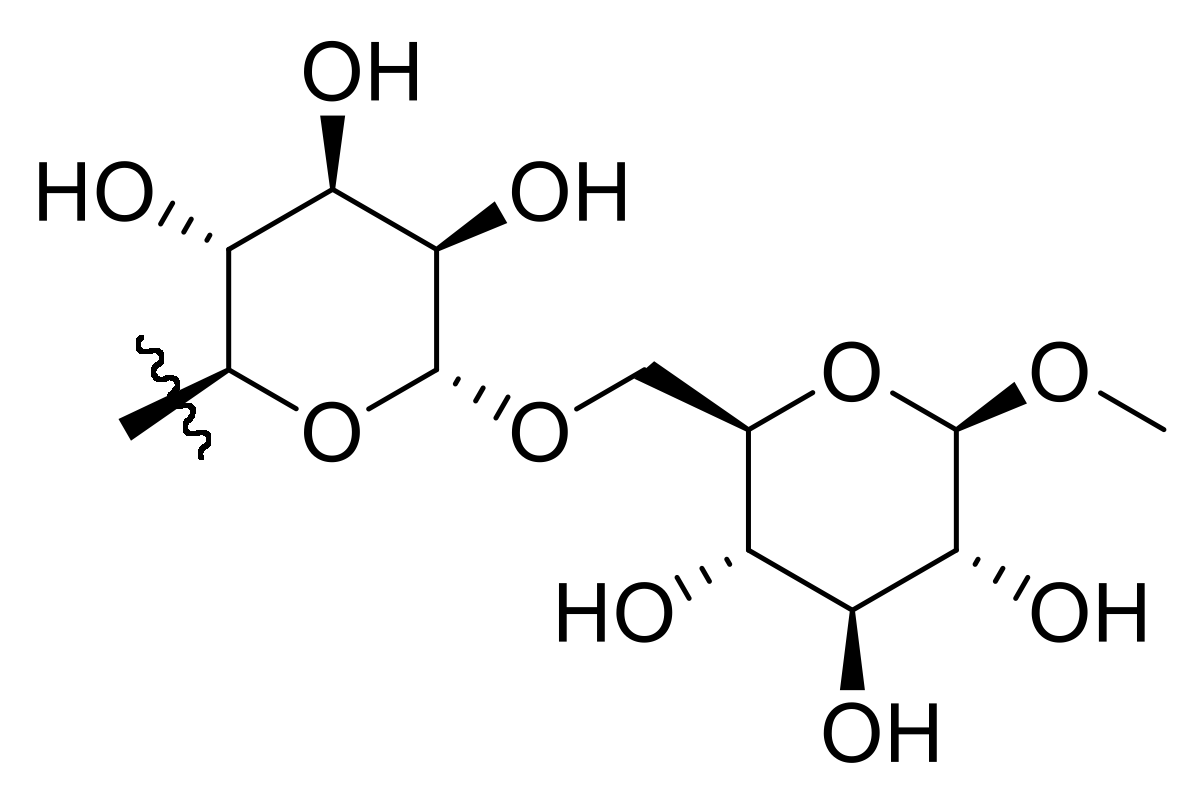 | H | OH | OCH_3_ | Hesperidin |
| **Entry** | **R_1_** | **R_2_** | **R_3_** | **R_4_** | **R_5_** | **Name** |
| 11 | OH |  | H | H | OH | Naringin |
| 12 | OH | OCH_3_ |  | H | H | Rotundaflavone Ia, Ib |
| 13 | OH | OH |  | H | H | Rotundaflavone IIa, IIb |

Flavones

| **Entry** | **R_1_** | **R_2_** | **R_3_** | **R_4_** | **R_5_** | **Name** |
| --- | --- | --- | --- | --- | --- | --- |
| 1 | OH | OH | H | H | H | 5,7-dihydroxyflavone |
| 2 | OH | OCH_3_ | H | OH | H | 5-hydroxy-7-methoxyflavone |
| 3 | OCH_3_ | OCH_3_ | H | H | OCH_3_ | 3,5,7-trimethoxyflavone |
| 4 | OH | OCH_3_ | OCH_3_ | OCH_3_ | OCH_3_ | 5-hydroxy-3,7,3’,4’-tetramethoxyflavone |
| 5 | OCH_3_ | OCH_3_ | OCH_3_ | OCH_3_ | H | 5,7,3’,4’-tetramethoxyflavone |
| 6 | OH | OCH_3_ | H | H | OCH_3_ | 5-hydroxy-3,7-dimethoxyflavone |
| 7 | OH | OCH_3_ | H | OCH_3_ | OCH_3_ | 5-hydroxy-3,7,4’-trimethoxyflavone |
| 8 | OH | OCH_3_ | H | OCH_3_ | H | 5-hydroxy-7,4’-dimethoxyflavone |
| 9 | OCH_3_ | OCH_3_ | H | H | H | 5,7-dimethoxyflavone |
| 10 | OCH_3_ | OCH_3_ | H | OCH_3_ | H | 5,7,4’-trimethoxyflavone |
| 11 | OCH_3_ | OCH_3_ | OCH_3_ | OCH_3_ | OCH_3_ | 3,5,7,3',4'-pentamethoxyflavone |
| 12 | OCH_3_ | OCH_3_ | H | OCH_3_ | OCH_3_ | 3,5,7,4’-tetramethoxyflavone |

Trihydroxyflavonols

| **Entry** | **R_1_** | **Name** |
| --- | --- | --- |
| 1 | OH | Quercetin |
| 2 | H | Kaempferol |

Chalcones

| **Entry** | **R_1_** | **R_2_** | **Name** |
| --- | --- | --- | --- |
| 1 |  | H | 2’,4’,6’-trihydroxychalcone (Pinocembrin Chalcone) |
| 2 |  | H | 2’,6’-dihydroxy-4’-methoxychalcone (Pinostrobin Chalcone) |
| 3 |  | H | 2’,4’-dihydroxy-6’-methoxychalcone (Cardamonin) |
| **Entry** | **R_1_** | **R_2_** | **Name** |
| 4 |  | OH | 2’,4,4’-trihydroxy-6’-methoxy-chalcone (Helichrysetin) |
| 5 |  | H | 2'-hydroxy-4',6'- dimethoxychalcone |
| 6 |  | OCH_3_ | 2'-hydroxy-4,4',6'-trimethoxychalcone |
| 7 |  | H | Rubranine |
| 8 |  | H | (±)-Boesenbergin A |
| 9 |  | H | (±)-Boesenbergin B |

Dihydrochalcones

| **Entry** | **R_1_** | **R_2_** | **R_3_** | **R_4_** | **R_5_** | **R_6_** | **Name** |
| --- | --- | --- | --- | --- | --- | --- | --- |
| 1 | OH | OH | OCH_3_ | OH | H | H | 4,2’,4’-trihydroxy-6’-methoxydihydrochalcone |
| 2 | OH | OH | OH | OH | H | H | 4,2’,4’,6’-tetrahydroxydihydrochalcone |
| 3 | H | H | H | OCH_3_ | OH | OH | 2,6-dihydroxy-4-methoxydihydrochalcone |
| 4 | OH | OH | OH | H | H | H | 2’,4’,6’-trihydroxydihydrochalcone (Propiophenone) |
| 5 | OH | OH | OCH_3_ | H | H | H | 2’,4’-diihydroxy-6’-methoxydihydrochalcone (Uvangoletin) |

Cinnamoyl devatives

| **Entry** | **R_1_** | **R_2_** | **R_3_** | **Name** |
| --- | --- | --- | --- | --- |
| 1 | H | H | OCH_3_ | Methyl cinnamate |
| 2 | OH | OH | OH | Caffeic acid |
| 3 | H | OH | OH | ρ-coumaric acid |
| 4 | OH | OH |  | Chlorogenic acid |
| 5 | H | H |  | Cinnamyl cinnamate |

Cyclohexenylchalcone derivatives

| **Entry** | **R_1_** | **R_2_** | **R_3_** | **R_4_** | **R_5_** | **Name** |
| --- | --- | --- | --- | --- | --- | --- |
| 1 | Ph |  |  | CH_3_ | H | (+)-4-Hydroxypanduratin A (1R,2R,3S);  (-)-4-Hydroxypanduratin A (1S,2S,3R) |
| 2 | Ph |  |  | CH_3_ | H | (+)-Panduratin A (1R,2R,3S);  (-)-Panduratin A (1S,2S,3R) |
| 3 | Ph |  |  | CH_3_ | H | (+)-Isopanduratin A (1R,2R,3S);  (-)-Isopanduratin A (1S,2S,3R) |
| 4 | Ph | 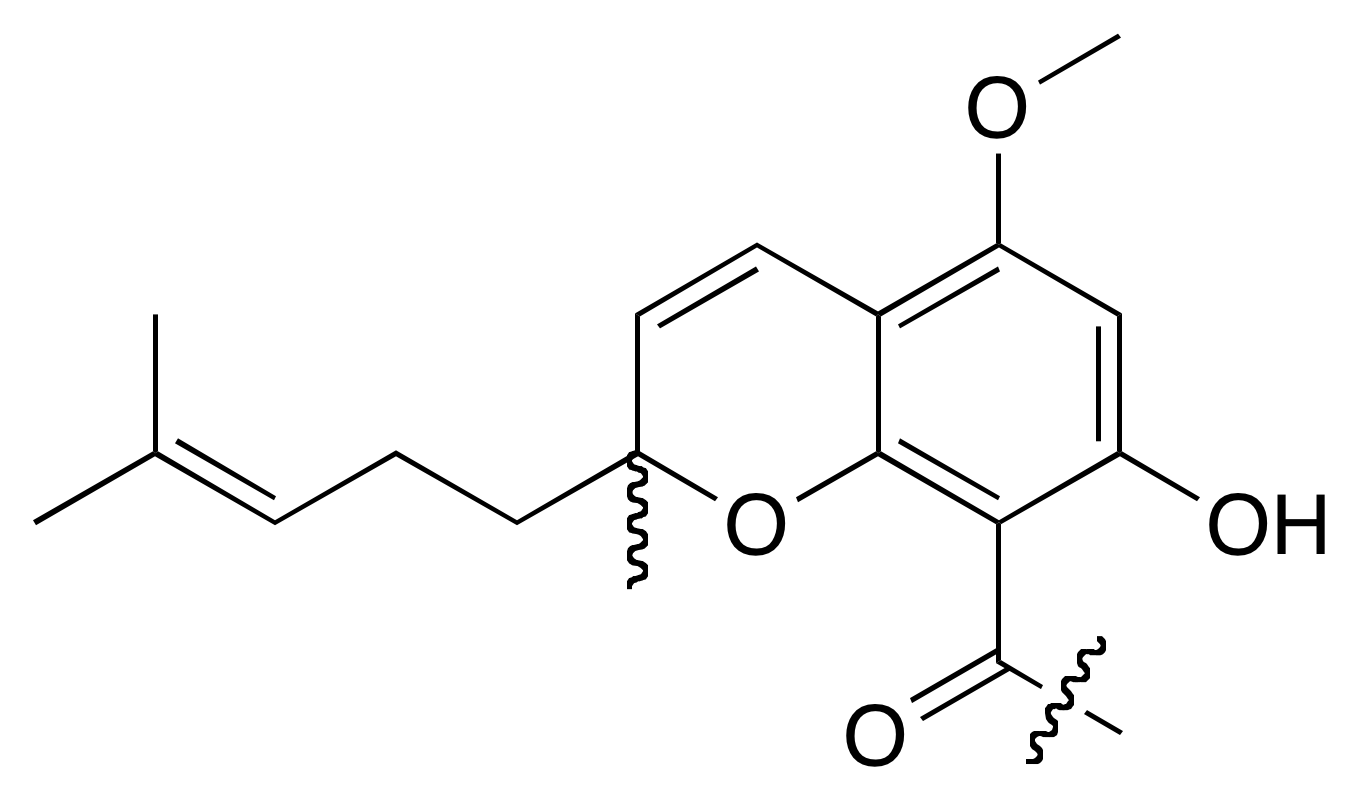 |  | CH_3_ | H | Panduratin B1  Panduraytin BII |
| 5 |  |  |  | CH_3_ | H | Panduratin C |
|  |  |  |  |  |  |  |
| **Entry** | **R_1_** | **R_2_** | **R_3_** | **R_4_** | **R_5_** | **Name** |
| 6 | Ph |  |  | CH_3_ | H | Panduratin D |
| 7 | Ph |  |  | CH_3_ | H | Panduratin E |
| 8 | Ph |  |  | CH_3_ | H | Panduratin F |
| 9 |  | Ph |  | CH_3_ | H | Panduratin G |
| 10 | Ph |  |  | CH_3_ | H | Panduratin H |
| 11 |  | Ph |  | CH_3_ | H | Panduratin I |
| 12 | Ph |  | H | H |  | (+)-Krachaizin A (1R,2R);  (-)-Krachaizin A (1S,2S) |
| **Entry** | **R_1_** | **R_2_** | **R_3_** | **R_4_** | **R_5_** | **Name** |
| 13 | Ph |  | H | H |  | (+)-Krachaizin B (1R,2R);  (-)-Krachaizin B (1S,2S) |

Esters

| **Entry** | | **R_1_** | | **R_2_** | **Name** | | | | |  |
| --- | --- | --- | --- | --- | --- | --- | --- | --- | --- | --- |
| 1 | | Ph | |  | Geranyl Benzoate | | | | |  |
| 2 | | C14H29 | | C4H9 | n-butyl-n-pentadecanoate | | | | |  |
| 3 | | CH_3_ | | C8H17 | Methyl-n-nonanoate | | | | |  |
| 4 | |  | | C6H13 | n-hexyl angelate | | | | |  |
| 5 | | C2H5 | |  | Trans-2-hexanyl-n-propionate | | | | |  |
| 6 | | CH_3_ | |  | Neryl acetate | | | | |  |
| 7 | | C2H5 | |  | Cyclohexyl-n-propionate | | | | |  |
| 8 | | Ph | | C2H5 | Ethyl benzoate | | | | |  |
| 9 | | C9H19 | |  | Guaiacol n-capraoate | | | | |  |
| 10 | | C4H9 | |  | 2-(4-methyl-1-cyclohex-3-enyl)propan-2-yl pentanoate (Terpinyl valerate) | | | | |  |
| **Entry** | **R_1_** | | **R_2_** | | | **R_3_** | **R_4_** | **R_5_** | **Name** | |
| 11 | | CH_3_ | |  | 2-cyclohexylethyl acetate | | | | |  |
| 12 | | H | |  | Geranyl formate | | | | |  |
| 13 | |  | | CH_3_ | 2,4-dihydroxy-6-phenethyl-benzoic acid methyl ester | | | | |  |
| 14 | |  | |  | Geranyl-2,4-dihydroxy-6-phenylbenzoate | | | | |  |
| 15 | | CH_3_ | |  | 2,2,2-trichloro-1-phenylethyl acetate (Rosephenone) | | | | |  |

Kawains

5,6-Dehydrokawain Dihydro-5,6-Dehydrokawain

Terpenes and Terpenoids

I. Acyclic

II. Cyclic

Miscellaneous
